# CMSV: Long-Read-Based Structural Variation Detection Through a CNN–Mamba Model

**DOI:** 10.3390/genes17060633

**Published:** 2026-05-30

**Authors:** Song Cheng, Hongbing Ma

**Affiliations:** 1 School of Computer Science and Technology, Xinjiang University, Urumqi 830046, China; 2 Department of Electronic Engineering, Tsinghua University, Beijing 100084, China

**Keywords:** structural variation, long-read sequencing, SV detection, Mamba, deep learning

## Abstract

**Background/Objectives:** Structural variations are important forms of genomic variation and are closely related to genomic diversity and many human diseases. Long-read sequencing has improved the ability to detect structural variations in complex genomic regions, but existing methods still mainly rely on manually designed heuristic rules and often have difficulty jointly modeling local SV signatures and cross-subsegment contextual modeling. To address this problem, we propose CMSV, a structural variation detection and genotyping method for long-read sequencing data. **Methods:** CMSV extracts multi-channel position-level features from alignment results and combines a multi-scale convolutional encoder with stacked Mamba modules for window-level candidate region detection. Candidate variants are then integrated and optimized through DBSCAN-based density clustering and length-based clustering. Genotypes are inferred based on variant-supporting reads and reference-genome-supporting reads. CMSV is designed to support several major structural variation types, including DEL, INS, DUP, INV, and TRA/BND. In our real-data benchmarks, the strongest validation is provided for DEL and INS, while DUP, INV, and TRA/BND are further evaluated using simulated multi-type datasets. **Results:** Experiments on real HG002 DEL/INS benchmarks, simulated multi-type datasets, and family-based datasets show that CMSV is competitive across PacBio CCS, PacBio CLR, and ONT platforms within the corresponding evaluation settings. Additional held-out chromosome evaluation on GRCh38 chr13–chr22 was further conducted to assess chromosome-level generalization beyond the full HG002 benchmark. CMSV shows stable performance in DEL/INS detection and genotyping on real-data benchmarks, while simulated multi-type evaluations further support its ability to detect DUP, INV, and TRA/BND. The results also show that CMSV can effectively model complex variant signals and maintain good family-level consistency in trio-based evaluation. **Conclusions:** CMSV provides an effective deep learning framework for long-read structural variation detection and genotyping across sequencing platforms and coverage levels.

## 1. Introduction

Structural variations (SVs) are an important source of genomic diversity. They usually refer to genomic rearrangement events with a length of at least 50 bp [1]. Major types include deletion (DEL), insertion (INS), inversion (INV), duplication (DUP), and translocation/breakend (TRA/BND). Compared with single-nucleotide variants [2] and short insertions/deletions [3], SVs often affect larger genomic regions [4]. They are also closely related to many human diseases [5,6,7], complex traits, and population-level genetic differences [4]. Therefore, accurate SV detection and genotyping are of great importance for understanding genomic structural variation, investigating disease mechanisms, and advancing clinical genomics research.

At present, SV detection mainly relies on two types of sequencing technologies: short-read sequencing [8] and long-read sequencing [9]. Short-read platforms have high base accuracy and low sequencing cost. As a result, many mature SV detection methods [10,11,12,13] have been developed for them. However, because of the limited read length, short-read data are often unable to span repetitive regions or cover large structural variants [14,15]. This greatly limits their detection sensitivity and breakpoint resolution in low-complexity regions and complex rearrangement scenarios [13,15,16]. In contrast, long-read sequencing platforms, such as Pacific Biosciences (PacBio), Menlo Park, CA, USA [17] and Oxford Nanopore Technologies (ONT), Oxford, UK [18], can generate long sequence reads that span repetitive regions and complete variant sites [19,20]. Therefore, they have clear advantages in the discovery and characterization of complex SVs.

Nevertheless, SV detection based on long-read data still faces many challenges. First, the relatively high error rate of long-read sequencing can obscure true breakpoint signals and introduce substantial noise into alignment results [19,20]. Second, current mainstream long-read SV detection tools, such as Sniffles2 (v2.3.1) [21], cuteSV2 (v2.0.3) [22], and SVIM (v2.0.0) [23], mainly rely on manually designed heuristic rules based on CIGAR patterns, split-read evidence, clipping signals, and local coverage changes [24]. These methods perform well in many standard scenarios. However, their performance may still decline markedly when sequencing noise is high, breakpoint patterns are ambiguous, or complex rearrangements are difficult to distinguish from alignment artifacts. In addition, heuristic rules are usually not sufficient to capture the heterogeneity of SV signals across different genomic regions. They are also not well suited for jointly modeling local SV evidence patterns and cross-subsegment contextual relationships across regions.

In recent years, deep learning has provided a new direction for long-read SV detection [12,25]. Convolutional neural networks (CNNs) [26,27,28] and attention mechanisms [29,30,31] have been introduced into genomic variant detection, and several long-read SV detection methods based on deep learning have been developed [32,33,34,35,36]. By learning feature representations directly from alignment-derived signals, deep learning models may capture complex patterns that are difficult for traditional rules to model explicitly. However, existing deep learning-based SV detection methods still have some limitations. For example, INSnet [32] and LSnet [37] focus only on a limited number of SV types (mainly INS or DEL) and cannot support multiple major variant categories within a unified framework. Some methods use image-based input representations [38,39]. This may lose the sequential structure of the original signals during transformation. Some other methods emphasize either local feature extraction or global context modeling [40,41,42], without fully exploiting the complementarity between the two. For long-read SV detection, a key question remains how to preserve the original sequence structure while effectively modeling both local SV evidence patterns and cross-subsegment contextual relationships within candidate regions.

To address the above issues, this paper proposes CMSV, a structural variation detection and genotyping method for long-read sequencing data. CMSV first extracts multi-channel genomic signal representations from alignment results and divides the genome into fixed-length windows. It then uses a multi-scale convolutional encoder [27] enhanced by Squeeze-and-Excitation modules [43] to capture local SV evidence patterns. After that, stacked Mamba modules [44] based on selective state space models [45] are applied to model the context of subsegment sequences within each window to perform window-level candidate region detection. On this basis, CMSV further combines CIGAR features and split-read evidence to extract candidate variants, performs DBSCAN-based density clustering [46] followed by length-based clustering and breakpoint refinement, and completes genotyping based on variant-supporting reads and reference-supporting reads. This framework mainly supports DEL, INS, DUP, INV, and translocation-related events represented as TRA/BND. It can also adapt to data from different long-read sequencing platforms and different sequencing depths.

## 2. Materials and Methods

### 2.1. Overview of the Method

**Figure 1:**
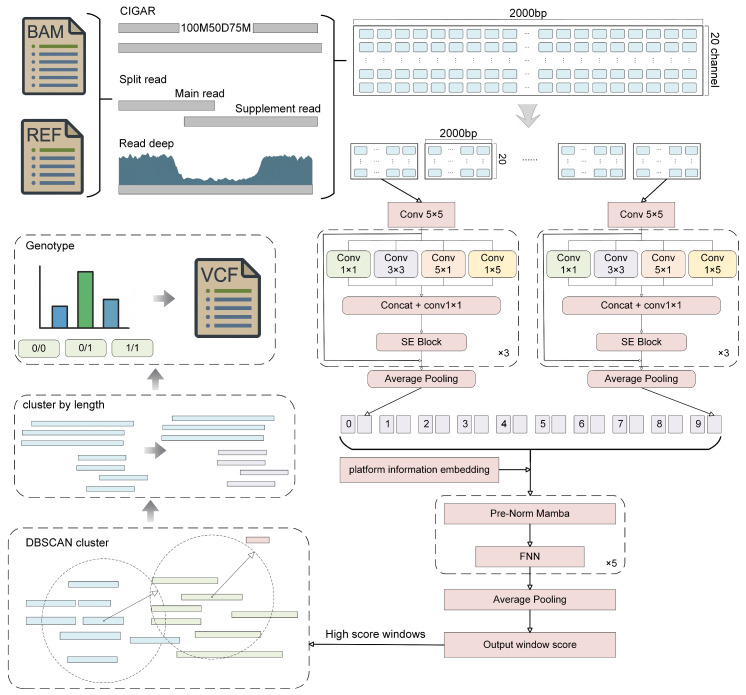
Overview of the CMSV framework. CMSV first extracts multi-channel position-level features from long-read alignments and constructs fixed-length windows as model inputs. It then uses a multi-scale convolutional encoder and stacked Mamba modules to perform high-recall window-level candidate region detection. Candidate regions are further converted into read-level candidate variants through CIGAR and split-read evidence. Candidate variants are then integrated by DBSCAN-based density clustering, length-based refinement, and TRA/BND-specific breakpoint grouping. Finally, CMSV performs genotyping based on variant-supporting reads and reference-supporting reads and outputs the results in standard VCF format.

CMSV is a structural variation (SV) detection tool designed for long-read sequencing data. Its goal is to improve the sensitivity of candidate region discovery and to achieve robust candidate clustering and genotyping. CMSV takes reads aligned to the reference genome as input. It extracts multi-channel feature signals to represent regions that may contain SVs. These signals are then organized into fixed-size window samples and fed into a framework composed of a multi-scale convolution (MC) encoder and a Mamba state space (MS) module for representation learning and classification, thereby identifying potential SV candidate regions. After that, candidate variants of the same SV type are clustered and integrated. Based on supporting read information, CMSV filters candidate variants and infers their genotypes. Finally, CMSV outputs the SV detection results in standard VCF format. Figure 1 shows the overall workflow of CMSV.

### 2.2. Input Representation and Sample Construction

CMSV first divides the reference genome into consecutive segments by chromosome. It then extracts multiple alignment-derived signals from the BAM file. These signals are used to characterize features related to structural variants (SVs) at the regional level. They include CIGAR alignment patterns, split-read breakpoint alignments, local coverage, and soft/hard clipping information. Some of these signals are strand-specific. To preserve this strand-specific information, CMSV represents signals from the forward strand and the reverse strand separately. This avoids distortion during feature construction. This design finally produces a 20-channel position-level feature matrix, with 10 channels for the forward strand and 10 channels for the reverse strand.

To reduce scale differences across channels, CMSV applies Z-score normalization to each channel within each genomic region, as follows:

$${\hat{x}}_{ij}=\frac{x_{ij}-\mu_{j}}{\sigma_{j}+\epsilon }$$

where μ_j_ and σ_j_ denote the mean and standard deviation of the j-th channel, respectively, and ϵ is a small constant added to ensure numerical stability. After normalization, the feature matrix is divided into fixed-length 2000 bp intervals for model training and prediction. Each interval is treated as an independent sample during both training and inference. More details on channel construction and sample generation are provided in the Supplementary Note S1 and Supplementary Table S1.

### 2.3. CNN–Mamba-Based Window-Level Candidate Region Detection

To capture both local SV features and cross-subsegment contextual modeling, CMSV adopts a CNN-Mamba framework for window-level candidate region detection. Each 2000 bp input window is divided by genomic coordinates into 10 consecutive and equal-length subsegments, and each subsegment has a length of 200 bp. First, a shared local encoder is applied to each subsegment to extract local features. Then, the resulting feature representations are fed into a sequence modeling module to determine whether the current input window contains a potential SV.

This hierarchical encoding strategy separates local pattern recognition from cross-subsegment context integration. The former mainly focuses on extracting discriminative local features from sparse, noisy, and highly heterogeneous alignment signals. The latter is used to model the compositional relationships of SV evidence patterns across different subsegments and their positional consistency.

The local encoder uses a multi-scale residual convolution structure with four branches for each subsegment. The four branches extract features with different receptive fields. They are: (1) a 1 × 1 pointwise convolution for modeling inter-channel relationships; (2) a 3 × 3 standard convolution for capturing short-range local sequence patterns within the subsegment; (3) a 5 × 1 dilated convolution with a dilation rate of 2 for capturing medium-range dependencies along the sequence dimension; and (4) a 1 × 5 convolution for modeling joint patterns across different signal types.

The outputs of the four branches are concatenated along the channel dimension. They are then passed through a squeeze-and-excitation (SE) module [43] for dynamic channel reweighting and fused through a residual connection to form the final local representation. This design improves the representation of breakpoint-related local patterns, coverage changes, and longer-range structural signals. After global average pooling, each subsegment is mapped to a 128-dimensional embedding vector. Therefore, each input window is finally represented as a sequence of 10 subsegment embeddings. Detailed configurations of the convolution layers and output dimensions are provided in the Supplementary Note S2 and Supplementary Table S2.

After obtaining the subsegment-level embeddings, the model further introduces learnable positional embeddings and platform embeddings. These are used to model the systematic error differences among the PacBio CCS, PacBio CLR, and ONT sequencing platforms. The resulting embedding sequence is then fed into five stacked residual Mamba modules for contextual modeling. Each module contains a Pre-Norm Mamba sublayer and a feed-forward network (FFN) sublayer. Both sublayers use residual connections. Mamba models the sequence in a state-space manner and has linear time complexity. It can therefore effectively capture cross-subsegment contextual relationships between subsegments while keeping the computational cost low. After contextual modeling, the representations of all subsegments are mean-pooled, and an SV classification head is used to output the probability that the current window belongs to an SV candidate region. More details on the number of layers, state dimensions, and FFN parameters are provided in the Supplementary Note S2 and Supplementary Table S3. The model was trained using the following hyperparameters and implementation settings, detailed in Supplementary Note S5 and Table S7.

### 2.4. Candidate Extraction and Clustering-Based Integration

After obtaining the window regions containing candidate variants, CMSV merges adjacent positive windows to form continuous candidate regions. For each candidate variant region, CMSV goes back to the original BAM file and extracts candidate variants based on CIGAR features and split-read breakpoint alignments. CIGAR features are mainly used to identify deletions (DEL) and insertions (INS) with a length of no less than 40 bp. Split-read evidence is mainly used to identify more complex variants, including inversions (INV), duplications (DUP), and translocations/breakends (TRA/BND).

After candidate extraction, CMSV integrates candidate variants by SV type. For deletion (DEL) and duplication (DUP) variants, CMSV uses DBSCAN clustering [46] and sets the clustering radius according to the SV type and the estimated alignment error range. For insertion (INS) and inversion (INV) variants, CMSV further introduces length-based radius stratification (Supplementary Note S4 and Supplementary Table S6) to reduce interference between variants of different lengths. Candidate variants are first divided into different groups by length. Each group is then assigned a corresponding clustering radius. Candidate variants that fail to form high-density clusters are retained as singleton candidates to avoid mistakenly discarding true variants with low support. For translocation/breakend (TRA/BND) variants, CMSV first groups candidate events according to chromosome-pairing relationships and then clusters and integrates them based on the proximity of breakpoints on both sides.

After DBSCAN-based clustering, CMSV further refines candidate variants through length-based clustering. This is done to avoid incorrectly merging variants that are close in genomic position but differ greatly in length. For each final subcluster, CMSV uses the median start position of its member variants as the representative breakpoint and the median variant length as the representative SV length. This strategy reduces the influence of outlier reads.

### 2.5. Genotyping

Candidate variants that pass the minimum-support filtering step enter the genotyping stage. This stage relies on two types of read-count evidence: variant-supporting reads and reference-supporting reads. Variant-supporting reads are reads that explicitly support the candidate variant through CIGAR or split-read evidence. The definition of reference-supporting reads varies across SV types. For INS and TRA/BND variants, a read is considered reference-supporting if it spans a sufficient region around the breakpoint and does not support the alternative event. For DEL, INV, and DUP variants, reference-supporting reads are defined based on continuous spanning coverage across the corresponding reference interval. Candidate variants with an effective number of supporting reads below the minimum support threshold are labeled as ungenotyped (./.) and are not subjected to likelihood comparison.

For each remaining candidate variant, CMSV compares the posterior scores of three standard diploid genotype states, {0/0, 0/1, 1/1}. Let a denote the number of variant-supporting reads, r denote the number of reference-supporting reads, and ϵ denote the effective sequencing/alignment error parameter. In the current implementation, ϵ is treated as a platform-level empirical parameter to account for sequencing and alignment uncertainty, rather than being estimated separately for each candidate variant. The genotype likelihoods are defined as

$$L(0/0)=(1-\epsilon {)}^{r}\cdot \epsilon^{a}$$

$$L(0/1)=[b\cdot (1-\epsilon ){]}^{r}\cdot [(1-b)\cdot (1-\epsilon ){]}^{a}$$

$$L(1/1)=\epsilon^{r}\cdot (1-\epsilon {)}^{a}$$

where b is a heterozygous balance factor, which is set to 0.5 by default. This default setting corresponds to a balanced heterozygous model, in which variant-supporting reads and reference-supporting reads are expected to contribute approximately equally. Values of b > 0.5 indicate a stronger contribution from reference-supporting reads under the heterozygous model, whereas values of b < 0.5 indicate a stronger contribution from variant-supporting reads. Therefore, the default value b = 0.5 should not be interpreted as introducing a reference-allele bias. A sensitivity analysis of b and per-genotype performance is provided in Supplementary Note S10 and Tables S9 and S10. Given the genotype priors P (0/0), P (0/1), and P (1/1), the posterior probability of genotype g is computed as

$$P(g|r,a)=\frac{L(g)\cdot P(g)}{\sum_{g^{\prime }}L(g^{\prime })\cdot P(g^{\prime })+\epsilon_{\mathrm{clamp}}}$$

where ϵ_clamp_ = 10^−10^ is a small constant used to avoid numerical underflow. CMSV uses a uniform prior by default, namely P (0/0) = P (0/1) = P (1/1) = 1/3. The genotype with the highest posterior score is selected as the final genotype. If r + a = 0, indicating that no effective read evidence is available near the candidate breakpoint, the genotype is directly assigned as (./.). In implementation, all likelihood and posterior score computations are performed in the log domain to ensure numerical stability.

## 3. Results

### 3.1. Benchmark on HG002 on hs37d5

**Figure 2:**
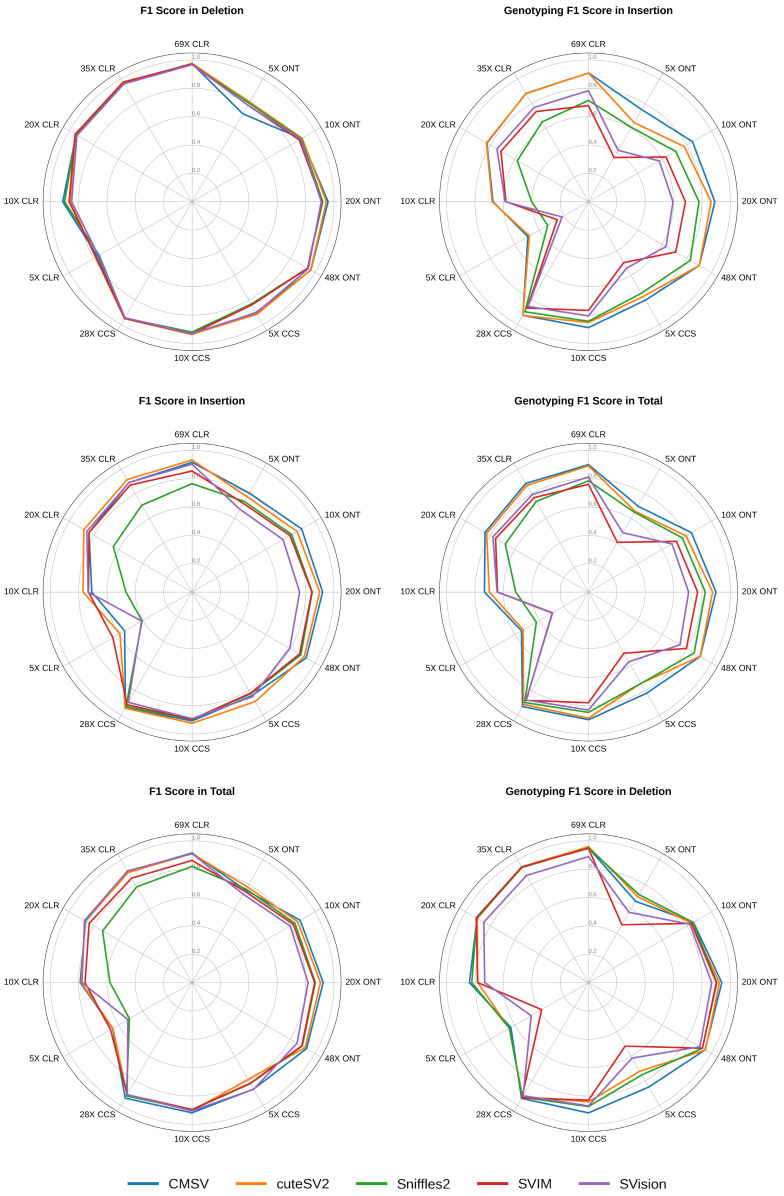
Detection and genotyping performance on hs37d5-aligned HG002 data. Comparison of CMSV with representative long-read SV callers across multiple sequencing depths on the PacBio CCS, PacBio CLR, and ONT platforms. Detection and genotyping results are summarized by overall F1 scores.

To evaluate the performance of CMSV on real long-read datasets, we conducted benchmark tests on the aligned data of the HG002 sample from Genome in a Bottle (GIAB), National Institute of Standards and Technology, Gaithersburg, MD, USA against the hs37d5 reference genome [47]. The data included multiple sequencing depths from the PacBio CCS, PacBio CLR, and ONT platforms. We compared CMSV with several representative long-read SV callers, including cuteSV2 [22], Sniffles2 [21], SVIM [23], and SVision (v1.4.0) [33] (Figure 2). These tools were selected because they are among the most widely used and most mature structural variation detection software packages for long-read sequencing. They have been extensively benchmarked on multiple datasets, show robust performance across different sequencing technologies, and are still actively maintained and updated. Because the high-confidence HG002 truth sets used in the real-data benchmarks mainly provide reliable evaluation for DEL and INS, the real-data results focus on these two SV types. The performance of DUP, INV, and TRA/BND is evaluated mainly in the simulated multi-type benchmark.

Across the three real long-read platforms, CMSV showed strong detection performance, although the results varied with platform and sequencing depth. On PacBio CCS data, CMSV achieved the highest overall detection F1 at 28× (94.22%) and 10× (91.87%). At 5× (86.77%), its result was also close to the best, indicating good robustness to reduced coverage. On PacBio CLR data, CMSV also remained highly competitive. It ranked first at 69× (91.48%) and 10× (78.85%) and was close to the best result at 35× (90.25%) and 5× (64.70%). On ONT data, CMSV achieved the best overall detection F1 at 48× (93.20%), 20× (92.40%), and 10× (87.97%). Overall, the real-data results show that CMSV is highly competitive across all three platforms, with the strongest overall performance on CCS and ONT data.

In genotyping, compared with the results at the detection level, CMSV showed a clearer and more stable advantage. On PacBio CCS data, it achieved the highest overall genotyping F1 at all evaluated depths: 28× (93.11%), 10× (89.97%), and 5× (82.17%). On PacBio CLR data, it also ranked first at all tested depths, with overall genotyping F1 scores of 89.94% at 69×, 88.50% at 35×, 84.24% at 20×, 73.35% at 10×, and 54.58% at 5×. A similar trend was also observed on ONT data, where CMSV achieved the highest overall genotyping F1 at every depth. These results support the reliability of CMSV in the hs37d5-based evaluation. Detailed per-coverage detection and genotyping metrics on the hs37d5 benchmark are provided in Supplementary Tables S11–S16.

### 3.2. Benchmark on HG002 on GRCh38

After evaluating with the HG002 truth set on hs37d5, we used the liftover tool to map the GIAB HG002 high-confidence truth set from hs37d5 to GRCh38. We also downloaded the corresponding alignment files and performed evaluations across different platforms and coverage depths in the same way as for hs37d5. The detailed data are provided in the Supplementary Materials, Supplementary Tables S17–S22.

For detection, on the PacBio CLR platform, CMSV obtained a higher total F1 score than any other tested variant detection method at all sequencing depths from 10× to 70× (10× = 74.61%, 20× = 87.10%, 35× = 90.79%, 70× = 92.99%). At 5× coverage on PacBio CLR, CMSV also performed well (55.62%), although SVIM achieved the highest F1 score (59.59%). CMSV obtained the highest F1 score at medium coverage (20×; 92.40%) and full coverage (93.02%) on ONT data, but at low coverage it was only slightly better than cuteSV2. On the PacBio CCS platform, CMSV was generally comparable with the other methods while still achieving the highest F1 performance, such as 92.29% at 10× coverage.

For genotyping, CMSV ranked first on PacBio CCS across the entire evaluated depth range (5× = 82.81%, 10× = 90.67%, full depth = 93.65%). It also ranked first at medium and high coverage on PacBio CLR sequencing data (10× = 68.20%, 20× = 83.14%, 70× = 90.92%), although it was slightly lower than cuteSV2 on the 5× CLR data (43.23% vs. 45.56%). On the ONT platform, CMSV remained competitive compared with Sniffles2 and SVIM, but cuteSV2 achieved the best overall genotyping performance. Overall, the GRCh38-based evaluation results show that CMSV remained highly competitive, especially in detection tasks on CLR and ONT data and in genotyping tasks on CCS and CLR data.

In addition, because the CMSV learning module was trained using HG002 chromosomes 1–10, we performed a separate held-out chromosome evaluation on GRCh38 chromosomes 13–22. Prior to the call-level benchmark, we examined the window-level recall of the CNN-Mamba detector and the threshold sensitivity of final calls (Supplementary Note S3 and Supplementary Tables S4 and S5). The results are reported in Supplementary Note S8 and Supplementary Tables S23–S28.

### 3.3. Benchmark on NA19240 and HG00514

To evaluate CMSV in a more challenging scenario, we conducted supplementary evaluations on two samples, HG00514 (Chinese Han, CHS, 1000 Genomes Project, European Bioinformatics Institute, Hinxton, UK) and NA19240 (Yoruba, YRI, 1000 Genomes Project, European Bioinformatics Institute, Hinxton, UK). The truth sets for these two samples were derived from an integrated analysis of multi-platform sequencing data, covering more complex genomic regions and containing more large-size variants, which made the evaluation more difficult. We divided SVs by length into five intervals: 50–200 bp, 200–500 bp, 500–1000 bp, 1000–5000 bp, and 5000+ bp, and evaluated DEL and INS separately. The results are shown in Table 1.

For DEL detection, CMSV achieved the highest F1 in all intervals above 200 bp in both samples (HG00514: 75.25%, 61.74%, 74.53%, 67.27%; NA19240: 75.77%, 64.53%, 74.70%, 71.52%). It was only slightly inferior to cuteSV2 in the 50–200 bp interval, with its advantage being particularly clear for medium- and large-size variants.

For INS detection, the advantage of CMSV was mainly observed in the medium- and large-size intervals. It ranked first in all intervals above 200 bp in both HG00514 and NA19240. In particular, in the 1000–5000 bp interval, CMSV achieved 51.69% on HG00514 and 45.81% on NA19240, clearly outperforming the second-best method, cuteSV2, which achieved 33.93% and 24.47%, respectively.

Overall, CMSV showed stable and clear advantages in detecting medium- and large-size structural variants. To further assess its robustness under low-coverage conditions, we also evaluated HG00514 and NA19240 at 10× and 5× coverage. Additional results are provided in Supplementary Tables S35 and S36, showing that CMSV generally maintained relatively stable performance when the sequencing coverage was reduced.

### 3.4. Benchmark on Simulated Data

**Figure 3:**
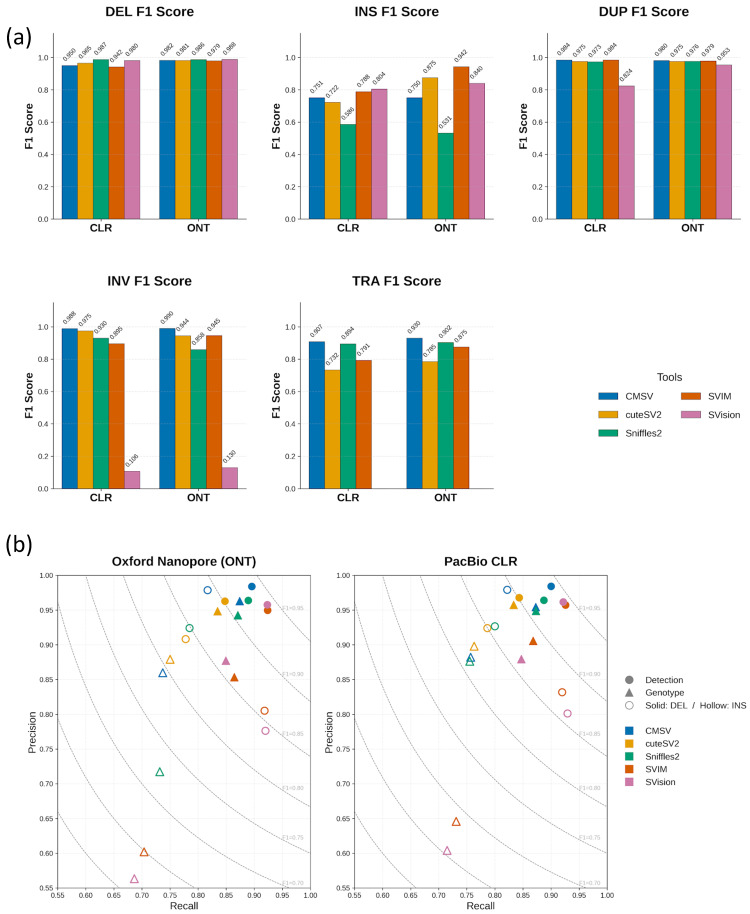
Benchmark performance on simulated SURVIVOR and VISOR datasets. Performance comparison of CMSV and other methods on two simulated benchmark datasets. (**a**) The SURVIVOR dataset includes five SV types and is mainly used to evaluate detection performance across different variant categories. (**b**) The VISOR dataset focuses on diploid DEL and INS events and supports both detection and genotyping evaluation.

To further evaluate the model, we conducted experiments on two simulated benchmark datasets. One benchmark was based on the SURVIVOR (v1.0.7) [48] simulated dataset, which contains five types of structural variants (SVs), namely deletion (DEL), insertion (INS), duplication (DUP), inversion (INV), and translocation (TRA). The other benchmark was based on the VISOR (v1.1.2) [49] simulated dataset. This dataset mainly focuses on DEL and INS under a diploid setting. It is therefore suitable for both detection evaluation and genotype-aware assessment. The simulation results are summarized in Figure 3.

On the SURVIVOR benchmark, the main advantage of CMSV lay in its ability to identify complex structural variants. On both the CLR and ONT platforms, CMSV achieved the highest F1 scores for INV and TRA detection. On CLR, the F1 scores reached 98.84% and 90.75%, respectively. On ONT, they reached 98.99% and 92.99%, respectively. In addition, CMSV also maintained leading or near-leading performance in DUP detection. This suggests that the method has a clear advantage in modeling complex rearrangement patterns. In contrast, CMSV was not the best in all settings for DEL, and especially for INS, although it still remained highly competitive overall. Benefiting from its strong performance on complex SV types, CMSV achieved the highest average F1 across the five SV types on the CLR platform and ranked second overall on the ONT platform. Overall, these results show that the core strength of CMSV on the SURVIVOR benchmark mainly comes from its effective modeling and robust identification of complex variants.

On the VISOR detection benchmark, CMSV showed a clear performance advantage in specific aspects. It achieved the highest INS detection F1 on both the CLR (89.37%) and ONT (89.05%) platforms, indicating a strong ability to detect insertion variants under diploid simulation settings. For DEL detection, CMSV also remained among the leading methods, with stable and competitive overall performance. Overall, the VISOR detection results show that CMSV is particularly strong in insertion detection while also maintaining solid overall performance in deletion detection.

In the VISOR genotyping task, the main advantage of CMSV was observed in DEL genotyping. It achieved the best results on both the CLR (91.17%) and ONT (91.64%) platforms, indicating strong stability and accuracy in genotyping deletion variants. For INS genotyping, CMSV also ranked among the top methods on both platforms. It was slightly below cuteSV2 but still remained one of the most competitive methods for this task. Taken together, the VISOR results show that CMSV has strong overall competitiveness under diploid simulation settings. Its detection advantage is more evident for insertion variants, while its genotyping advantage is more evident for deletion variants. Complete simulated benchmark results by SV type, platform, and evaluation mode are provided in Supplementary Note S6 and Supplementary Tables S29–S34.

### 3.5. Trio-Based Evaluation

**Figure 4:**
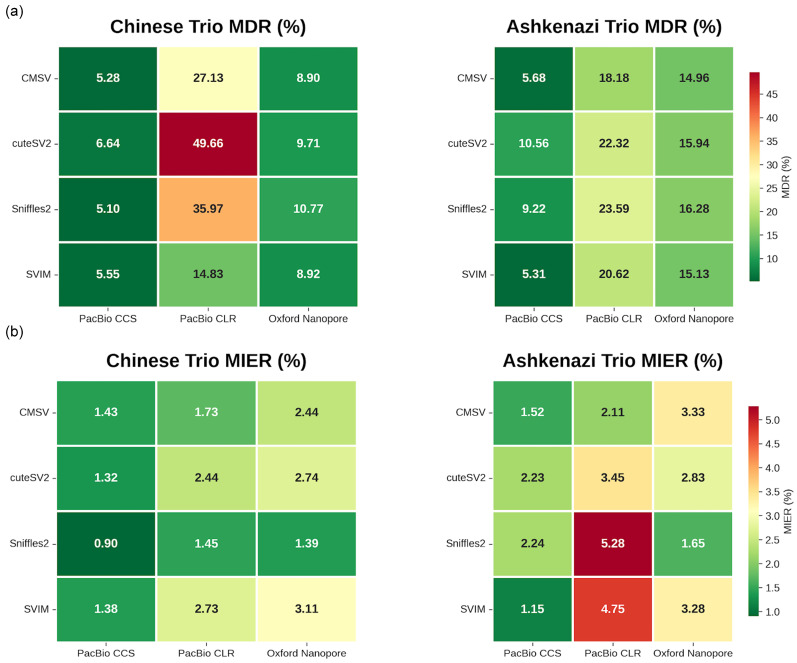
Family-based evaluation on real trio datasets. (**a**) Comparison of different SV callers on the Chinese and Ashkenazi trio datasets using the Mendelian discordance rate (MDR). MDR measures the proportion of child SV calls without matched parental support based on presence/absence concordance. (**b**) Comparison of different SV callers using the Mendelian inheritance error rate (MIER). MIER provides a complementary genotype-aware measure calculated on matched trio records with available genotypes in all three family members. Lower MDR and MIER values indicate better family-level consistency. Detailed SV-type-stratified MDR and MIER results are provided in the Supplementary Materials.

Mendelian inheritance provides an important basis for evaluating the consistency of inherited variants. It has also been widely used to assess SV detection tools. To evaluate the detection consistency and reliability of different methods in parent–child samples, we used two real trio datasets: the Ashkenazi trio and the Chinese trio. First, we calculated the Mendelian discordance rate (MDR) at the presence/absence level. For each SV detected in the offspring sample, we searched for a matched variant in the paternal and maternal samples. The match was determined according to predefined parent–child matching criteria. If no matched variant was found in either parent, this SV was counted as a Mendelian-discordant variant. A lower MDR indicates better family-level consistency. We compared CMSV with SVIM, Sniffles2, and cuteSV2. The evaluation covered three sequencing platforms: PacBio CCS, PacBio CLR, and Oxford Nanopore. The results are shown in Figure 4a.

For the Chinese trio, CMSV achieved the lowest MDR on the Oxford Nanopore platform, with a value of 8.90%. This was slightly lower than SVIM at 8.92%. It was also clearly lower than cuteSV2 at 9.71% and Sniffles2 at 10.77%. On the PacBio CCS platform, CMSV had an MDR of 5.28%. This was slightly higher than Sniffles2 at 5.10%, but lower than SVIM at 5.55% and cuteSV2 at 6.64%. On the PacBio CLR platform, CMSV had an MDR of 27.13%. This was higher than SVIM at 14.83%, but clearly lower than Sniffles2 at 35.97% and cuteSV2 at 49.66%. Therefore, in the Chinese trio, CMSV performed best on Oxford Nanopore and remained competitive on PacBio CCS. However, it was less favorable than SVIM on PacBio CLR. This suggests that its family-level consistency on CLR data still has room for improvement.

For the Ashkenazi trio, CMSV also showed strong family-level consistency across the three platforms. On the Oxford Nanopore platform, CMSV achieved the lowest MDR, with a value of 14.96%. This was slightly lower than SVIM at 15.13%, cuteSV2 at 15.94%, and Sniffles2 at 16.28%. On the PacBio CLR platform, CMSV also achieved the lowest MDR, with a value of 18.18%. This was lower than SVIM at 20.62%, cuteSV2 at 22.32%, and Sniffles2 at 23.59%. On the PacBio CCS platform, CMSV had an MDR of 5.68%. This was slightly higher than SVIM at 5.31%, but clearly lower than cuteSV2 at 10.56% and Sniffles2 at 9.22%. These results show that, in the Ashkenazi trio, CMSV achieved good family-level consistency on Oxford Nanopore and PacBio CLR. It also remained highly competitive on PacBio CCS.

To further support the trio analysis, we also calculated the Mendelian inheritance error rate (MIER) as a supplementary genotype-aware metric, as shown in Figure 4b. It should be noted that MIER is different from the presence/absence-based MDR. MDR focuses on whether an offspring SV lacks matched support from either parent. In contrast, MIER is calculated on matched trio records for which genotype information is available in all three family members. A record is considered Mendelian-consistent only when the offspring genotype satisfies diploid inheritance rules given the genotypes of both parents. Therefore, MDR and MIER reflect different aspects of trio consistency, and their absolute values should not be directly compared. Detailed MDR and MIER results stratified by SV type are provided in Supplementary Tables S37–S40.

The MIER results show that CMSV maintained low genotype-level Mendelian inconsistency among matched trio records. For the Chinese trio, the MIER values of CMSV were 1.43%, 1.73%, and 2.44% on PacBio CCS, PacBio CLR, and Oxford Nanopore, respectively. For the Ashkenazi trio, the MIER values of CMSV were 1.52%, 2.11%, and 3.33% on PacBio CCS, PacBio CLR, and Oxford Nanopore, respectively. These results indicate that, among matched trio records with complete genotype information, most offspring genotypes predicted by CMSV followed the Mendelian genotype combinations allowed by the parental genotypes.

### 3.6. Ablation Study

To evaluate the contribution of different feature modeling modules to SV detection performance, we compared three model variants: CNN-only, Mamba-only, and CNN-Mamba. The CNN-only model uses only the convolutional encoding branch to extract local pattern features from the input feature matrix. The Mamba-only model uses only the sequence modeling branch to capture cross-subsegment contextual relationships between subsegments. The CNN-Mamba model first uses CNN to extract local pattern features and then uses Mamba to model contextual information in the complete feature sequence. During training, all models were trained using the same data split and evaluation pipeline. Chromosomes 1 to 10 were used as the training set. Chromosomes 11 and 12 were used as the validation set. Chromosomes 13 to 22 were used as the test set. The number of training epochs was fixed at 30 for all models.

**Figure 5:**
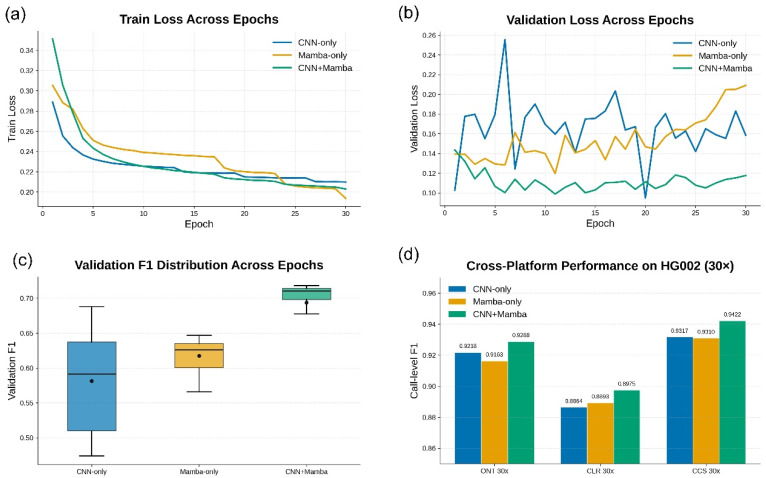
Ablation study of the CNN–Mamba architecture. (**a**) Training loss curves of the three model variants; (**b**) validation loss curves of the three model variants; (**c**) distribution of validation F1 scores of the three model variants; and (**d**) cross-platform comparison of call-level F1 for the three model variants on HG002 data at 30× coverage.

The training dynamics show clear differences among the three architectures in both fitting ability and validation behavior. As shown in Figure 5a, the Mamba-only model achieved the fastest decrease in training loss and the lowest final training error, indicating a strong ability to fit the training objective. However, this advantage did not lead to better performance on the validation set. As shown in Figure 5b, the validation loss of the Mamba-only model rose markedly in the later stage of training. The CNN-only model reached a low validation loss early in training, but its validation loss fluctuated greatly in the later stage, indicating poor stability. In contrast, the CNN-Mamba model maintained a more stable validation loss throughout the whole training process. This suggests that combining local convolutional features with sequence-level context modeling helps improve the validation behavior of the model.

The epoch-wise validation F1 distribution showed a similar trend. As shown in Figure 5c, the CNN-Mamba model achieved both the highest upper bound and the highest median validation F1 among the three models. Its more compact boxplot distribution indicates that this performance gain did not come from occasional fluctuations at a few training time points. Instead, it showed stronger discriminative ability throughout most stages of training. These results suggest that the local discriminative features captured by CNN and the cross-subsegment contextual information modeled by Mamba are clearly complementary. As a result, the model achieved higher peak validation performance and more favorable validation dynamics.

To examine whether these conclusions still hold on real data, we further conducted a full cross-platform evaluation on HG002 data at 30× coverage, and the results are shown in Figure 5d. On all three platforms, namely ONT, CLR, and CCS, CNN-Mamba achieved the highest call-level F1. On the ONT and CCS platforms, CNN-only performed better than Mamba-only. On the CLR platform, Mamba-only was slightly better than CNN-only. These results indicate that convolutional modeling and sequence-level context modeling have different strengths on different platforms. However, their combination leads to the best overall performance. Overall, CNN-Mamba performed best across all three platforms, showing that the complementarity between local convolutional modeling and cross-subsegment contextual modeling improves the reliability and robustness of the model.

## 4. Discussion

This study proposes CMSV, a structural variation detection and genotyping method for long-read sequencing data. CMSV encodes alignment-derived evidence signals into multi-channel position-level features. It combines a multi-scale convolutional encoder with stacked Mamba modules to jointly model local SV evidence patterns and cross-subsegment contextual modeling, thereby enabling window-level candidate region detection. On this basis, CMSV further combines CIGAR features and split-read evidence to perform candidate variant extraction, clustering, and integration, breakpoint refinement, and genotyping. This framework supports major SV types, including DEL, INS, DUP, INV, and TRA/BND. It also shows good applicability and robustness across different long-read sequencing platforms and sequencing depths.

Compared with traditional long-read SV detection methods, CMSV has three main advantages. First, existing heuristic methods are often limited in high-noise and complex rearrangement scenarios. In contrast, CMSV combines multi-scale convolution and Mamba modeling. It can capture both local discriminative patterns and cross-subsegment contextual relationships and, thus, represent complex variant signals more effectively. Second, CMSV directly builds position-level feature representations from alignment-derived signals, rather than converting them into image inputs. This helps preserve the original sequence structure and at the same time improves the characterization of multiple types of SV evidence. Third, in the candidate integration stage, CMSV adopts DBSCAN-based density clustering followed by length-based refinement and further combines breakpoint refinement with a genotyping module, which improves the stability of the final results. An extended comparison with other CNN-based hybrid deep learning SV callers (INSnet, MAMnet, SVHunter) under the same evaluation setting is provided in Supplementary Note S9 and Supplement Table S8.

The experimental results verify the effectiveness of the above design. In real-data evaluation, CMSV showed strong competitiveness on the PacBio CCS, PacBio CLR, and ONT platforms. Its overall detection performance was particularly strong on CCS and ONT data. In the genotyping task, the advantage of CMSV was that it was more stable. This indicates that it can not only effectively identify candidate variants but also maintain high reliability in subsequent genotype inference. On simulated data, CMSV showed strong performance on complex SVs such as INV and TRA in the SURVIVOR benchmark. The VISOR benchmark showed clear advantages in INS detection and DEL genotyping. Family-based evaluation showed that CMSV achieved strong Mendelian consistency on ONT and competitive consistency on CCS, whereas its MDR on CLR was higher than that of SVIM. The ablation results further indicated that multi-scale convolution and Mamba modeling provide complementary contributions to CMSV. In addition, the full HG002 benchmark should be interpreted as a partial in-sample comparison because CMSV was developed using HG002-derived chromosome-level training data. Therefore, the held-out chr13–chr22 evaluation, external-sample HG00514/NA19240 benchmarks, trio-consistency analysis, and simulated evaluations provide complementary evidence for generalization.

Although CMSV showed strong performance in multiple evaluation settings, it still has some limitations. Compared with lightweight heuristic SV callers, CMSV has higher computational and memory overhead because its full workflow includes multiple stages, such as feature generation, model inference, candidate extraction, clustering and integration, and genotyping. As shown in Table S41, under the tested settings, CMSV required longer runtime and higher peak host memory than Sniffles2, cuteSV2, and SVIM, but it was faster than SVision. In the current implementation, the main bottleneck comes from feature generation and the storage/loading of intermediate .npy feature files, because alignment-derived signals must first be converted into multi-channel window-level representations before CNN-Mamba inference. The model inference step requires a CUDA-enabled GPU in the current implementation, whereas clustering and post-processing account for a smaller proportion of the total runtime. Therefore, CMSV is currently more suitable for accuracy-oriented analysis than for ultra-fast large-cohort screening. Future work will focus on streaming feature construction, reducing intermediate .npy storage, and improving inference efficiency.

In addition, although CMSV remains competitive under low-coverage conditions, there is still room for further improvement in extremely low-coverage scenarios. This is also reflected in the benchmark results, where the advantage of CMSV became less consistent in low-coverage CLR and ONT settings, especially for genotyping. Reduced read support and platform-specific alignment noise may affect candidate integration, allele-balance estimation, and genotype assignment. The current real-data benchmarks mainly provide strong validation for DEL and INS, whereas the evaluation of DUP, INV, and TRA/BND relies more on simulated multi-type datasets and trio-consistency analyses. Therefore, the results for complex SV types should be interpreted more cautiously, especially for categories such as DUP and BND, where real-data truth sets and family-based consistency remain more challenging. Further evaluation on more comprehensive, real complex-SV truth sets is still needed. The current method is also mainly designed for germline SV detection. It has not yet been specifically designed for somatic variants, nested SVs, or more complex rearrangement structures.

## Figures and Tables

**Table 1:** F1 score comparison (DEL/INS) by SV size bin on HG00514 and NA19240.

Dataset	Bin	CMSV (DEL/INS)	SVIM (DEL/INS)	cuteSV2 (DEL/INS)	Sniffles2 (DEL/INS)
HG00514	50–200	0.2504/0.3258	0.1362/0.2074	0.3265/0.4424	0.1608/0.2911
	200–500	0.7525/0.4545	0.0432/0.0303	0.6296/0.4741	0.5746/0.3912
	500–1000	0.6174/0.2601	0.1245/0.0358	0.3962/0.2231	0.4609/0.2423
	1000–5000	0.7453/0.5169	0.1627/0.0134	0.5601/0.3393	0.6650/0.0886
	5000+	0.6727/0.2385	0.1871/0.0000	0.5239/0.0940	0.6275/0.0149
NA19240	50–200	0.2677/0.2920	0.1327/0.1693	0.3279/0.4089	0.1701/0.2800
	200–500	0.7577/0.4654	0.0371/0.0245	0.5664/0.4116	0.5818/0.4023
	500–1000	0.6453/0.2604	0.1154/0.0396	0.3877/0.2053	0.4537/0.2353
	1000–5000	0.7470/0.4581	0.1639/0.0056	0.4503/0.2447	0.6467/0.0704
	5000+	0.7152/0.0808	0.1798/0.0000	0.4569/0.0324	0.6524/0.0051

## Data Availability

The HG002 Tier1 benchmark structural variant call set and high-confidence regions are publicly available from the GIAB consortium at https://ftp.ncbi.nih.gov/giab/ftp/data/AshkenazimTrio/analysis/NIST_SVs_Integration_v0.6/ (accessed on 9 May 2026). Sequencing reads and BAM files for the Ashkenazim trio (HG002, HG003, and HG004) are publicly available at https://ftp.ncbi.nih.gov/giab/ftp/data/AshkenazimTrio/ (accessed on 9 May 2026), and sequencing reads and BAM files for the Chinese trio (HG005, HG006, and HG007) are publicly available at https://ftp.ncbi.nih.gov/giab/ftp/data/ChineseTrio/ (accessed on 9 May 2026). The PacBio BAM alignment file and the truth variant call set for sample HG00514 (Chinese Han, CHS) are publicly available at http://ftp.1000genomes.ebi.ac.uk/vol1/ftp/data_collections/hgsv_sv_discovery/working/20160905_smithm_pacbio_aligns/HG00514_bwamem_GRCh38DH_CHS_20160905_pacbio.bam (accessed on 9 May 2026) and https://ftp.ncbi.nlm.nih.gov/pub/dbVar/data/Homo_sapiens/by_study/genotype/nstd152/HG00514.BIP-unified.vcf.gz (accessed on 9 May 2026), respectively. For sample NA19240 (Yoruba, YRI), the PacBio BAM alignment file and the truth variant call set are publicly available at http://ftp.1000genomes.ebi.ac.uk/vol1/ftp/data_collections/hgsv_sv_discovery/working/20160905_smithm_pacbio_aligns/NA19240_bwamem_GRCh38DH_YRI_20160905_pacbio.bam (accessed on 9 May 2026) and https://ftp.ncbi.nlm.nih.gov/pub/dbVar/data/Homo_sapiens/by_study/genotype/nstd152/NA19240.BIP-unified.vcf.gz (accessed on 9 May 2026), respectively. The source code and pretrained model weights of CMSV are publicly available at https://github.com/cmsv-code/CMSV (accessed on 9 May 2026).

## Supplementary Materials

The following supporting information can be downloaded at: https://www.mdpi.com/article/10.3390/genes17060633/s1. Figure S1: Schematic comparison of CNN-based hybrid deep learning SV calling frameworks; Table S1: Definition of channel features; Table S2: CNN encoder architecture; Table S3: Mamba sequence modeling and classification head architecture; Table S4: Window-level performance on held-out HG002 CCS chr13–chr22; Table S5: Threshold sensitivity of final SV calls on HG002 CCS chr13–chr22; Table S6: Length-stratified DBSCAN eps parameters for INS and INV; Table S7: Default training hyperparameters of CMSV; Table S8: Comparison of CNN-based hybrid SV callers; Table S9: Sensitivity analysis of the heterozygous balance factor b for CMSV genotyping; Table S10: Per-genotype performance at the default setting b = 0.5; Table S11: Benchmark Results of detection on HG002 PacBio CCS Dataset (hs37d5); Table S12: Benchmark Results of detection on HG002 PacBio CLR Dataset (hs37d5); Table S13: Benchmark Results of detection on HG002 Oxford Nanopore Dataset (hs37d5); Table S14: Benchmark Results of Genotyping on HG002 PacBio CCS Dataset (hs37d5); Table S15: Benchmark Results of Genotyping on HG002 PacBio CLR Dataset (hs37d5); Table S16: Benchmark Results of Genotyping on HG002 Oxford Nanopore Dataset (hs37d5); Table S17: Benchmark Results of detection on HG002 PacBio CCS Dataset (GRCh38); Table S18: Benchmark Results of detection on HG002 PacBio CLR Dataset (GRCh38); Table S19: Benchmark Results of detection on HG002 ONT Dataset (GRCh38); Table S20: Benchmark Results of Genotyping on HG002 PacBio CCS Dataset (GRCh38); Table S21: Benchmark Results of Genotyping on HG002 PacBio CLR Dataset (GRCh38); Table S22: Benchmark Results of Genotyping on HG002 ONT Dataset (GRCh38); Table S23: Benchmark Results of detection on HG002 PacBio CCS Dataset (GRCh38 chr13-chr22); Table S24: Benchmark Results of detection on HG002 PacBio CLR Dataset (GRCh38 chr13-chr22); Table S25: Benchmark Results of detection on HG002 Dataset (GRCh38 chr13-chr22); Table S26: Benchmark Results of Genotyping on HG002 PacBio CCS Dataset (GRCh38 chr13-chr22); Table S27: Benchmark Results of Genotyping on HG002 PacBio CLR Dataset (GRCh38 chr13-chr22); Table S28: Benchmark Results of Genotyping on HG002 ONT Dataset (GRCh38 chr13-chr22); Table S29: Benchmark results on SURVIVOR simulated CLR detection; Table S30: Benchmark results on SURVIVOR simulated ONT detection; Table S31: Benchmark results on VISOR simulated CLR detection; Table S32: Benchmark results on VISOR simulated ONT detection; Table S33: Benchmark results on VISOR simulated CLR genotyping; Table S34: Benchmark results on VISOR simulated ONT genotyping; Table S35: Detection performance on HG00514 and NA19240 datasets at 10× coverage; Table S36: Detection performance on HG00514 and NA19240 datasets at 5× coverage; Table S37: Mendelian discordance rate (MDR) results stratified by SV type in the Ashkenazi trio; Table S38: Mendelian discordance rate (MDR) results stratified by SV type in the Chinese trio; Table S39: Mendelian-inheritance error rate comparison on the GIAB Ashkenazi Jewish trio; Table S40: Mendelian-inheritance error rate comparison on the GIAB Chinese human trio; Table S41: Resource consumption of different SV callers. The Supplementary File includes detailed feature-channel definitions, model settings, dataset information, and benchmark results across sequencing platforms, coverage levels, SV types, and evaluation settings.

## Abbreviations

SV	Structural variation
DEL	Deletion
INS	Insertion
INV	Inversion
DUP	Duplication
TRA	Translocation
BND	Breakend
CCS	Circular consensus sequencing
CLR	Continuous long read
ONT	Oxford Nanopore Technologies
CNN	Convolutional neural network
SE	Squeeze-and-excitation
DBSCAN	Density-based spatial clustering of applications with noise
MDR MIER	Mendelian discordance rate Mendelian Inheritance Error Rate
GIAB	Genome in a Bottle
VCF	Variant call format
BAM	Binary alignment map
