# Supplementary material for: CMSV: Long-Read-Based Structural Variation Detection Through a CNN–Mamba Model
Source: Genes (Basel). 2026 May 30;17(6):633. doi: 10.3390/genes17060633 (PMC13300244; doi:10.3390/genes17060633)
Supplement: Supplementary file 1 [file genes-17-00633-s001.zip › genes-4337649-supplementary.pdf]

Supplementary Material

Supplementary Note S1. Details of input representation

CMSV directly constructs multi-channel feature vectors for each reference genomic position from BAM alignments. For each covered reference position, the model accumulates 20 signal channels. These channels are divided into two symmetric groups according to the alignment strand of reads: ten channels for the reverse strand (channels 0–9) and ten channels for the forward strand (channels 10–19). This strand-separated design preserves direction-related breakpoint evidence. It also prevents such evidence from being smoothed out by strand-agnostic aggregation. This is especially useful for distinguishing variants with strand-asymmetric patterns, such as inversions and translocations, from one-sided alignment artifacts. For the ten channels on each strand, the first two channels record position counts of CIGAR deletion operators (D) and insertion operators (I). Only operators with a length of at least 40 bp are counted during feature accumulation. The next five channels record different types of split-read breakpoint evidence. They correspond to DEL, INS, INV, DUP, and BND. The eighth channel records read depth on the corresponding strand. It is accumulated from the reference genomic positions covered by primary alignments. The last two channels record soft- and hard-clipping signals at both ends of reads. They correspond to the SM pattern, which represents clipping at the 5′ end, and the MS pattern, which represents clipping at the 3′ end. The complete list and meaning of all channels are provided in Table S1. The feature accumulation process only uses primary alignments that pass standard quality filtering. Unmapped, duplicate, secondary, and supplementary alignments are excluded. This avoids counting the same

breakpoint evidence more than once between primary and supplementary alignments.

To reduce redundancy caused by the high error rate of long-read sequencing, CMSV applies length-adaptive merging to CIGAR-derived variants during feature accumulation. For CIGAR DEL, nearby deletion operators are merged within a fixed 150 bp window. The start coordinate of the merged variant is set to the leftmost position, and the length is set to the accumulated length. For CIGAR INS, CMSV uses length-adaptive merging windows. Variants shorter than 100 bp are merged within a 200 bp window. Variants of 100–500 bp are merged within a 400 bp window. Variants longer than 500 bp are merged within a 600 bp window.

Split-read evidence is extracted by parsing reads with SA tags. CMSV compares the differences between primary and supplementary alignments in reference coordinates and read coordinates. The resulting variants are classified as DEL, INS, INV, DUP, or BND. To reduce noise from low-quality alignments, all split-read variants generally require MAPQ ≥ 20. For long INS variants with a length of at least 1000 bp, supplementary alignments are often shorter and have lower MAPQ values. Therefore, the MAPQ threshold is relaxed to MAPQ ≥ 10 for long INS variants. This avoids losing sensitivity for long insertions. For short INV variants shorter than 5000 bp, CMSV applies additional constraints besides strand reversal. The read-level segment gap must be no more than 500 bp, and the overlap must be no more than 200 bp. This helps remove short-range inversion false positives from local repetitive regions.

After feature accumulation, the feature matrix of each chromosome is divided into non-overlapping 2000 bp windows. Each window is used as a basic model input unit. Channel-wise Z-score normalization is applied independently within each window. The mean and standard deviation inside the window are used as the normalization reference. A small constant is added

to the standard deviation to avoid numerical instability in sparse-coverage regions with near-zero variance. The normalized windows and their start-coordinate indices are saved in .npy format. They are then loaded through a unified interface during training and inference. This interface hides differences in chromosome, sequencing platform, and coverage source. As a result, the same model can be trained and evaluated across multiple platforms and coverage levels.

Table S1. Definition of channel features

Channel	Channel name	Signal definition	Strand
0	del_cigar_rev	Position count of CIGAR deletion variants (≥ 40 bp; merged within adjacent 150 bp)	Reverse
1	ins_cigar_rev	Position count of CIGAR insertion variants (≥ 40 bp)	Reverse
2	del_split_rev	Position count of split-read DEL signals	Reverse
3	ins_split_rev	Position count of split-read INS signals	Reverse
4	inv_split_rev	Position count of split-read INV signals	Reverse
5	dup_split_rev	Position count of split-read DUP signals	Reverse
6	bnd_split_rev	Position count of split-read BND signals	Reverse
7	depth_rev	Read depth on the reverse strand (reference genomic position count)	Reverse
8	clip_sm_rev	Position count of 5′-end soft/hard clipping signals (SM pattern)	Reverse
9	clip_ms_rev	Position count of 3′-end soft/hard clipping signals (MS pattern)	Reverse
10–19	Corresponding forward-strand channels	Definitions symmetric to channels 0–9, derived from forward-strand reads	Forward

Supplementary Note S2. CNN–Mamba encoder architecture

Each 2000 bp input window is represented as a two-dimensional feature matrix with a shape of (2000, 20). The first dimension indexes genomic positions, and the second dimension indexes the 20 feature channels. To separate local pattern recognition from cross-region context modeling, CMSV divides each window along the genomic direction into 10 consecutive

subsegments of equal length. Each subsegment covers 200 bp and has a local feature representation of (200, 20).

The model has two stages. First, a shared multi-scale convolutional encoder independently encodes each subsegment and produces a fixed-dimensional subsegment embedding. Then, a stack of residual Mamba modules performs cross-subsegment contextual modeling among the 10 subsegment embeddings within the same window. The model finally outputs the probability that the window contains an SV.

The multi-scale convolutional encoder uses an early downsampling strategy. The stem layer downsamples the genomic dimension with a stride of 4. This allows the later multi-scale residual blocks to run at a lower resolution and reduces inference latency. The stem layer is a single Conv2d(1→32) layer with a kernel size of 5, a stride of (4, 1), and padding of 2. It is followed by BatchNorm2d and GELU activation. This layer compresses each subsegment from (200,20) to (50, 20) and increases the channel number to 32.

The stem layer is followed by one multi-scale residual block that increases the channel dimension from 32 to 64. Then, a downsampling convolution layer, Conv2d(64→64, kernel size = (5,1), stride = (5,1)), further reduces the sequence dimension from 50 to 10. Two additional multi-scale residual blocks are then applied at the (10, 20) resolution, and the number of output channels is kept at 128. Finally, AdaptiveAvgPool2d(1) summarizes each subsegment into a 128-dimensional embedding vector. The encoder parameters are shared across the 10 subsegments within the same window. The full encoder architecture and output shapes are listed in Table S2.

Each multi-scale residual block contains four parallel convolutional branches. These branches

are designed for different receptive fields and feature-axis interactions. The first branch is a 1×1 pointwise convolution. It models relationships among the 20 input channels without changing the spatial size. The second branch is a standard 3×3 convolution. It captures short-range local patterns along both the genomic and feature dimensions. The third branch is a (5,1) dilated convolution with a dilation rate of 2 along the genomic direction. Its effective receptive field reaches 9 bp along the sequence dimension, and it is used to model medium-range dependencies. The fourth branch is a (1,5) convolution. It operates along the feature-channel direction and explicitly models joint patterns among different signal channels.

The outputs of the four branches are concatenated along the channel dimension. Each branch contributes one quarter of the output channels. The concatenated features are then passed through a 1×1 projection convolution, BatchNorm2d, and GELU activation to restore the target channel dimension. A Squeeze-and-Excitation module with a reduction ratio of 8 is then used to recalibrate channel responses based on global average-pooling statistics. The result is added to the shortcut residual branch. When the input and output channel numbers differ, the shortcut branch uses a 1×1 convolution to align the dimensions.

The 10 subsegment embeddings produced by the convolutional encoder are organized as a sequence of length 10 for each window. Before entering the Mamba stack, two types of learnable conditional signals are added. The first is a learnable positional embedding with a shape of (1, 10, 128). It marks the relative position of each subsegment within the window and is initialized from a truncated normal distribution with a standard deviation of 0.02. The second is a learnable platform embedding with a shape of (3,128). It is also initialized from a truncated normal distribution with a standard deviation of 0.02.

The platform embedding is selected according to the sequencing platform ID of the current sample. ID 0 corresponds to PacBio CCS, ID 1 corresponds to PacBio CLR, and ID 2 corresponds to ONT. The selected platform embedding is broadcast and added to each subsegment embedding. This allows downstream layers to perform platform-conditioned computation based on systematic error patterns. The platform ID is automatically inferred during data generation from keywords in the BAM path, including ccs, hifi, clr, ont, and nanopore. It is then passed together with the feature tensor during training and inference.

Table S2. CNN encoder architecture

Layer	Operation	Output shape	Description
Stem	Conv2d(1→32, k=5, stride=(4,1), pad=2) + BN + GELU	(B, 32, 50, 20)	Early 4× downsampling along the sequence dimension
Block1	MultiScaleResBlock(32→64)	(B, 64, 50, 20)	Four branches + SE + residual connection
Down1	Conv2d(64→64, k=(5,1), stride=(5,1)) + BN + GELU	(B, 64, 10, 20)	5× downsampling along the sequence dimension
Block2	MultiScaleResBlock(64→128)	(B, 128, 10, 20)	Four branches + SE + residual connection
Block3	MultiScaleResBlock(128→128)	(B, 128, 10, 20)	Four branches + SE + residual connection
Pool	AdaptiveAvgPool2d(1) + Flatten	(B, 128)	Global pooling

The sequence modeling module consists of five stacked residual Mamba blocks. All Mamba blocks operate on 128-dimensional embeddings. Each block uses a pre-norm structure. LayerNorm is applied before each of the two sublayers, and the output of each sublayer is added back to the input through a residual connection. The first sublayer is a Mamba selective state space module. It uses a state dimension of 16, a convolution width of 4, and an expansion factor of 2. The second sublayer is a position-wise feed-forward network (FFN). Its hidden dimension is 512, which is four times the model dimension. It uses GELU activation and applies dropout

of 0.3 to both the hidden and output layers.

Table S3. Mamba sequence modeling and classification head architecture

Layer	Operation	Output shape	Description
Input	Subsegment embedding + positional embedding + platform embedding	(B, 10, 128)	Sequence of 10 subsegments
Block1	LN → Mamba(d_state=16, d_conv=4, expand=2) → Dropout(0.3) + Res → LN → FFN(128→512→128, GELU, Dropout=0.3) + Res	(B, 10, 128)	Pre-norm residual block
Block2	Same as Block1	(B, 10, 128)	Pre-norm residual block
Block3	Same as Block1	(B, 10, 128)	Pre-norm residual block
Block4	Same as Block1	(B, 10, 128)	Pre-norm residual block
Block5	Same as Block1	(B, 10, 128)	Pre-norm residual block
Norm	LayerNorm	(B, 10, 128)	Output normalization
Pool	MeanPool along the sequence dimension	(B, 128)	Window-level representation
Classifier	Linear(128→128) + ReLU + Dropout(0.4) + Linear(128→1) + Sigmoid	(B, 1)	Window-level SV probability

Mamba is used as a selective state-space sequence module to perform cross-subsegment contextual aggregation within each window. This property fits the sparse and asymmetric distribution of SV breakpoint signals within a window. After the five residual Mamba blocks, the output is passed through another LayerNorm. The 10 subsegment embeddings are then mean-pooled along the sequence dimension to obtain a 128-dimensional window-level representation. This representation is passed to a two-layer classification head: Linear(128→128), ReLU, Dropout(0.4), Linear(128→1), and Sigmoid. The final output is the

probability that the current window contains an SV breakpoint.

Supplementary Note S3. Decomposition analysis of the CNN–Mamba window detector

To further clarify the division of roles between the learning-based module in CMSV and the subsequent read-level candidate extraction and clustering procedures, we independently evaluated the CNN–Mamba window detector before candidate variant extraction and clustering. This analysis was performed on HG002 chr13–22, which were not used for model training or validation. For each 2000 bp window, the model outputs a candidate-region score. When this score is higher than the default threshold of 0.88, the window is classified as a candidate SV region.

In the window-level evaluation, a predicted positive window was counted as a true-positive window if it overlapped a true SV region. A predicted positive window that did not overlap any true SV region was counted as a false-positive window. A true SV region that was not covered by any positive window was counted as a false-negative event. In addition to window-level Precision, Recall, and F1, we also calculated the SV retention rate. This metric represents the proportion of true SVs covered by at least one positive candidate window before read-level candidate extraction.

As shown in Table S4, the CNN–Mamba window detector achieved high window-level recall across different sequencing platforms and retained most true SVs before read-level candidate extraction. For example, on HG002 CCS chr13–22, when the default threshold was set to 0.88,

the window-level recall reached 0.9575 and the SV retention rate reached 0.9813. This indicates that the learning-based window detector can effectively reduce the risk of missing true SV regions during the early candidate-region screening stage.

Table S4. Window-level performance on held-out HG002 CCS chr13–22

Dataset	Threshold	Window Precision	Window Recall	Window F1	SV retention rate
CCS chr13–22	0.88	0.2412	0.9575	0.3853	0.9813
ONT chr13–22	0.88	0.1780	0.9759	0.3010	0.9835
CLR chr13–22	0.88	0.1693	0.9736	0.2884	0.9799

It should be noted that the relatively low window-level precision is consistent with the design objective of this module. The CNN–Mamba window detector is not intended to directly generate final SV calls. Instead, it is used to screen SV-enriched candidate regions with high recall. In CMSV, final SV calls are generated after CIGAR/split-read candidate event extraction, DBSCAN clustering, length-consistency refinement, breakpoint summarization, and read-support filtering. Therefore, the CNN–Mamba module and the subsequent rule-based evidence integration module are not alternatives to each other, but are complementary components. The former learns candidate regions that may contain SVs from multi-channel alignment signals, whereas the latter further converts these candidate regions into precise read-level SV calls.

Table S5. Threshold sensitivity of final SV calls on HG002 CCS chr13–22

Threshold	Call count	Precision	Recall	F1
0.70	2754	0.9314	0.9454	0.9384
0.80	2753	0.9313	0.9451	0.9382
0.88	2749	0.9357	0.9426	0.9391
0.90	2749	0.9312	0.9436	0.9374
0.95	2741	0.9325	0.9421	0.9373

We further analyzed the sensitivity of final SV calling results to the window-score threshold.

In this experiment, the model weights and the downstream calling pipeline were kept unchanged, and only the candidate-window selection threshold was varied. The results are shown in Table S5.

As shown in Table S5, when the window-score threshold was varied from 0.70 to 0.95, the final F1 score remained stable between 0.9373 and 0.9391, and the call count changed only slightly. This indicates that the final SV calling results of CMSV do not depend on a finely tuned window threshold. Instead, the CNN–Mamba module provides a robust high-recall candidate-region screening step, while the subsequent read-level evidence extraction and clustering integration modules further determine the final SV calls. Overall, these results support the interpretation of CMSV as a learning-guided hybrid SV calling framework. In this framework, structural variation detection is achieved by combining neural representation learning with rule-based genomic evidence integration, rather than by relying on a single neural network classifier to directly output the final results.

Supplementary Note S4. Candidate variant clustering and length-based refinement

Read-level records extracted from candidate variants usually form dense clusters around the same breakpoint along the reference coordinates. Clear gaps are often observed between different true variants. CMSV therefore uses a staged clustering strategy. It first applies DBSCAN density clustering based on spatial position to form initial clusters. It then further splits each cluster by variant length. Finally, each subcluster is summarized into a single

representative variant using median values. This strategy considers both coordinate proximity and length consistency. It avoids incorrectly merging nearby true variants. It also avoids splitting true variants with sparse supporting evidence into multiple independent records.

The DBSCAN neighborhood parameter eps is selected according to both SV type and the average sequencing error rate. For DEL and DUP variants, CMSV uses fixed eps values based on error_mean. When error_mean > 0.1, which corresponds to high-error platforms such as CLR and ONT, the eps value is set to 1000 for DEL and 500 for both DUP and INV. When error_mean ≤ 0.1, which corresponds to low-error platforms such as CCS, eps = 1500 is used for all two types. The min_samples value is fixed at 2 for all SV types. This allows paired breakpoint evidence to form a cluster. This strategy provides a similar clustering granularity across platforms with different candidate-variant densities.

Table S6. Length-stratified DBSCAN eps parameters for INS and INV

SV type	Length range (bp)	Noisy eps (bp)	Clean eps (bp)	Noisy condition
INS	≤ 300	150	220	error_mean > 0.1
INS	301–1000	220	300	Same as above
INS	1001–5000	300	420	Same as above
INS	> 5000	420	600	Same as above
INV	≤ 3000	280	360	error_mean > 0.1
INV	3001–10000	420	520	Same as above
INV	> 10000	650	800	Same as above

For INS and INV, true variants with different length scales may occur near the same coordinate. A single eps value may cause short and long variants to interfere with each other. Therefore, CMSV further introduces a length-stratified adaptive eps strategy (Table S6). Candidate variants are first divided into several groups according to variant length. DBSCAN is then applied independently within each length group. The eps values for each group are divided into

noisy and clean settings according to the error level. Longer variants are allowed to use larger neighborhood radii to tolerate greater breakpoint estimation errors. Candidate variants that fail to form dense clusters are retained as singleton candidates. This prevents true variants with low support from being directly discarded.

After DBSCAN-based spatial clustering, CMSV applies the length-based secondary splitting function cluster_by_length to each multi-member cluster. The threshold ratio is set to 0.7. If the length variation coefficient within a subcluster exceeds this threshold, the subcluster is further split based on length similarity. This step is used to handle variants that are close in position but differ greatly in length. One example is the coexistence of short insertions and medium-length insertions in the same tandem repeat region.

For INS subclusters, CMSV further applies _trim_ins_length_outliers before summarization to remove extreme length outliers. If the tail length is more than twice the cluster median and differs by more than 30 bp, it is iteratively removed from the cluster until the length distribution becomes stable. This process reduces the influence of a single abnormally long read on the final variant length. It does not affect the main length estimate.

TRA/BND candidate variants do not enter the DBSCAN workflow. They are processed by an independent function, cluster_translocations. This function first groups candidate variants by chromosome pairing and direction. Variants in the same group are merged only when the reference-coordinate distances of both breakpoints are no more than 1000 bp. The representative breakpoints of the merged variant are defined by the median breakpoint coordinates of its members. The read-name list is defined as the union of member read names and is used as the supporting-read set.

For each integrated candidate variant, CMSV finally uses the median start coordinate of its members as the representative breakpoint and the median member length as the representative variant length. This reduces the influence of individual outlier reads on the final representative position and length.

Supplementary Note S5. Training and implementation details

CMSV training data consist of .npy feature files and their corresponding label files. The feature files are saved as 2000 bp windows during data generation. Each feature file is accompanied by an index file named _index.npy. The label files, named _label.npy, are generated automatically according to the overlap between the window indices and the truth VCF or truth BED files. If a window overlaps with any true SV, its label is set to 1. Otherwise, its label is set to 0. To reduce the severe class imbalance in SV detection, negative samples are randomly downsampled in each training split with neg_ratio = 4. This gives a final positive-to-negative ratio of about 1:4. A fixed random seed is used for negative sampling (seed = 42).

CMSV was trained as a single shared model across CCS, CLR, and ONT data, rather than as separate platform-specific models. Platform embeddings were used to provide platform-specific information to the shared CNN–Mamba model. We used 5× HG002 CLR, ONT, and CCS datasets, together with 5× CLR and ONT simulated datasets generated by SURVIVOR, for mixed training. The training, validation, and test sets were strictly separated by chromosome to avoid data leakage. Specifically, chromosomes 1–10 were used for training, chromosomes 11–12 were used for validation, and chromosomes 13–22 were used for testing. The sequencing platform of each sample was automatically inferred from keywords in the feature file path. Files

containing ccs or hifi were assigned to the CCS platform (platform_id = 0). Files containing clr were assigned to the CLR platform (platform_id = 1). Files containing ont or nanopore were assigned to the ONT platform (platform_id = 2). The platform ID was passed to the model together with the feature tensor during training and inference. It was used to drive the platform embedding layer for platform-conditioned computation.

Table S7. Default training hyperparameters of CMSV

Parameter	Value	Description
Optimizer	AdamW	Weight decay = 0.01
Initial learning rate	3×10-4	Target learning rate
Learning rate scheduler	ReduceLROnPlateau	factor = 0.5, patience = 5
Warmup epochs	3	Linear warmup
Number of training epochs	30	Total number of training epochs
Loss function	Binary Cross Entropy	Window-level binary classification
Negative sampling ratio	neg_ratio = 4	Positive-to-negative ratio of about 1:4
Random seed	42	Reproducible negative sampling
Dropout	0.3 / 0.4	Mamba blocks / classification head
Training chromosomes	chr1–chr10	Training data split
Validation chromosomes	chr11–chr12	Validation data split
Test chromosomes	chr13–chr22	Test data split

The model was trained with the AdamW optimizer. The initial learning rate was set to 3×10^{-4}, and the weight decay was set to 0.01. To reduce gradient instability at the beginning of training, a linear warmup strategy was used for the first 3 epochs. The learning rate was increased linearly from 0 to 3×10^{-4}. After warmup, a ReduceLROnPlateau scheduler was used to adjust the learning rate according to the validation loss. If the validation loss did not improve for 5 consecutive epochs, the learning rate was multiplied by 0.5. The total number of training epochs was 30. Binary cross-entropy was used as the classification loss. It was computed directly between the window-level SV probability predicted by the model and the binary label. All

training and inference experiments were conducted on NVIDIA CUDA-enabled GPUs. The model was implemented in PyTorch. The Mamba modules used efficient CUDA kernels provided by mamba_ssm and causal_conv1d. The default values of all key hyperparameters are summarized in Table S7.

Supplementary Note S6. Description of simulated datasets

To systematically evaluate the detection and genotyping performance of CMSV under controlled conditions, we used two simulated datasets: SURVIVOR and VISOR. SURVIVOR generates structural variants under a haploid setting. It is suitable for large-scale detection evaluation across five SV types. VISOR generates variants under a diploid setting and retains genotype information. It is therefore suitable for evaluating genotyping performance under controlled conditions. Together, these two datasets cover both SV type diversity and genotype resolution.

The SURVIVOR simulated dataset includes five SV types: DEL, INS, DUP, INV, and TRA. The complete parameter configuration is as follows:

```
DUPLICATION_minimum_length:     100
DUPLICATION_maximum_length:     50000
DUPLICATION_number:             1000
INDEL_minimum_length:           50
INDEL_maximum_length:           10000
INDEL_number:                   2000
TRANSLOCATION_minimum_length:   1000
TRANSLOCATION_maximum_length:   10000
TRANSLOCATION_number:           1000
INVERSION_minimum_length:       1000
```

```
INVERSION_maximum_length:       100000
INVERSION_number:               1000
INV_del_number:                 0
INV_dup_number:                 0
Number_haploid:                 1
homozygous_ratio:               0.6
```

On the variant references generated by SURVIVOR, long-read sequencing data were simulated using PBSIM. This was done to cover the typical error profiles of CLR and ONT platforms. For the CLR platform, reads were simulated with a base accuracy of 85%, a mean read length of 15 kb, and an error composition of 27% mismatches, 43% insertions, and 30% deletions. For the ONT platform, reads were simulated with a base accuracy of 92%, a mean read length of 15 kb, and an error composition of 12% mismatches, 75% insertions, and 13% deletions. The generated raw reads were aligned back to the reference genome using minimap2. Two coverage levels, 30× and 5×, were retained for evaluation. The SURVIVOR simulated data were evaluated using the SURVIVOR eval subcommand. The breakpoint distance tolerance was set to 500 bp.

The VISOR simulated dataset was constructed by following the simulation-based evaluation strategy used in existing long-read SV detection studies. It was used to evaluate the detection and genotyping ability of CMSV under a diploid setting. In this study, the VISOR experiment focused on two major SV types, DEL and INS. It retained the diploid genotype information of each variant and was therefore suitable for genotype-aware evaluation. We constructed 30× VISOR simulated datasets for both CLR and ONT platforms. These datasets contained 6976 DEL variants and 9668 INS variants in total. The VISOR dataset was evaluated using an in-house script.

Supplementary Note S7. Real-data evaluation

In the real-data evaluation, CMSV was systematically compared with four representative long-read SV detection tools: Sniffles2, cuteSV2, SVIM, and SVision. For cuteSV2, and SVision, which require an explicit setting of the minimum number of supporting reads, this study adjusted the minimum supporting-read parameter according to different sequencing depths. This was done to avoid artificially reducing their recall under low-coverage conditions due to overly high default thresholds. Specifically, for CLR and ONT data, the minimum number of supporting reads was set to 10 when the coverage was greater than 40×. For 35×, 20×, 10×, and 5× coverage, the values were set to 5, 4, 3, and 2, respectively. For CCS data, the values were set to 3, 2, and 1 for 28×, 10×, and 5× coverage, respectively. Sniffles2 and CMSV both support automatic selection of the minimum number of supporting reads, so no manual adjustment was needed. It should be noted that SVIM first generates a VCF file and then requires post-filtering of the resulting calls. The filtering thresholds used for SVIM were set according to the same rules as those used for cuteSV2 and SVision. The software versions used in this study were cuteSV2 v2.0.3, SVIM v2.0.0, Sniffles2 v2.3.1, and SVision v1.4.0 Because the inference commands varied slightly across sequencing platforms, we provide the commands for the PacBio CCS 28× dataset as representative examples.

```
cuteSV2:
cuteSV \
./input/HG002_CCS_28x.bam \
./reference/GRCh38.fa \
./output/cuteSV2/calls.vcf \
./output/cuteSV2/work \
-S HG002 \
```

```
-t 16 \
--genotype \
-s 3 \
-l 50 \
-md 500 \
-mi 500 \
--max_cluster_bias_INS 1000 \
--diff_ratio_merging_INS 0.9 \
--max_cluster_bias_DEL 1000 \
--diff_ratio_merging_DEL 0.5
```

Sniffles2:

```
sniffles \
  --input ./input/HG002_CCS_28x.bam \
  --vcf ./output/Sniffles2/calls.vcf.gz \
  --threads 16 \
  --minsupport auto \
  --minsvlen 50 \
  --reference ./reference/GRCh38.fa \
  --tandem-repeats ./annotation/human_GRCh38.trf.bed \
  --allow-overwrite
```

SVision:

```
svision \
  OMP_NUM_THREADS=16 \
  MKL_NUM_THREADS=16 \
  OPENBLAS_NUM_THREADS=16 \
  NUMEXPR_NUM_THREADS=16 \
  SVision \
    -o ./output/SVision/work \
    -b ./input/HG002_CCS_28x.bam \
    -m ./models/SVision/svision-cnn-model.ckpt \
    -g ./reference/GRCh38.fa \
```

```
      -n HG002_CCS_28x \
  -s 3 \
  SVIM:
  svim \
    ./output/SVIM/work \
    ./input/HG002_CCS_28x.bam \
    ./reference/GRCh38.fa \
    --min_sv_size 50 \
    --skip_genotyping \
    --symbolic_alleles
```

The real-data evaluation was performed using Truvari v4.1.0. The evaluation was restricted to the HG002 Tier1 v0.6 high-confidence regions using the --includebed option. Therefore, calls outside the high-confidence regions were not manually removed before evaluation, but were handled by the --includebed option during Truvari benchmarking. The main command was as follows:

```
  truvari bench \
      -b truth.vcf.gz \
      -c calls.vcf.gz \
      -o bench_out \
      --includebed HG002_SVs_Tier1_v0.6.bed \
      -r 1000 \
      -P 0 \
      --passonly
```

We used -r 1000 as the main breakpoint-distance tolerance. This is because different long-read SV callers may produce breakpoint position differences when reporting SV calls, especially for insertions and larger SVs, due to differences in variant representation and local alignment uncertainty. Therefore, this setting was used to reduce unfair penalties caused by small breakpoint representation differences, rather than treating these differences directly as true

biological discrepancies.

We used -P 0 in the primary evaluation to disable sequence-similarity matching. This is because not all callers can consistently output sequence-resolved insertion alleles. Some tools output symbolic insertions, whereas others output insertions with explicit inserted sequences. Therefore, enabling sequence-similarity matching could unfairly penalize callers that output symbolic insertion records. Under this setting, symbolic insertion records and sequence-resolved insertion records were evaluated using the same position-based matching strategy.

In the family-based evaluation, CMSV was compared with other methods on two real trio datasets: the Ashkenazi trio (HG002, HG003, and HG004) and the Chinese trio (HG005, HG006, and HG007). We used the Mendelian discordance rate (MDR) to evaluate the detection consistency of different methods in family samples. The basic principle is as follows. Under the normal Mendelian inheritance assumption, most germline structural variations detected in the offspring sample should have corresponding variants in either the paternal or maternal sample. Therefore, for each SV detected in the offspring sample, this study searched the callsets of both parents to determine whether a matched variant existed. If no matched variant was found in either parent, the variant was counted as a Mendelian-discordant variant. Finally, MDR was defined as the ratio of the number of Mendelian-discordant variants to the total number of SVs detected in the offspring sample. A lower MDR indicates better consistency between the SV callset generated by the method and family inheritance patterns.

In addition to MDR, we further calculated the Mendelian inheritance error rate (MIER) to evaluate family consistency with genotype information. Unlike MDR, which is based on whether a matched variant is present in either parent, MIER was calculated on matched trio

records for which genotype information was available in the offspring and both parents. For each matched trio record, we checked whether the offspring genotype followed diploid Mendelian inheritance rules given the genotypes of both parents. If the offspring genotype did not satisfy Mendelian inheritance under the parental genotypes, the record was counted as a Mendelian inheritance error. MIER was defined as the ratio of Mendelian inheritance errors to the total number of genotype-available matched trio records.

Supplementary Note S8. Chromosome-held-out evaluation on GRCh38 chromosomes 13–22

To further evaluate the generalization ability of CMSV on unseen chromosomes, we performed an additional evaluation of CMSV and the baseline methods on HG002 chr13–22 aligned to the GRCh38 reference genome (Tables S22–S27). This setting is more challenging than the standard HG002 benchmark used in the main experiments, because chr13–22 were not used for model training or validation. Therefore, this evaluation can be used to examine chromosome-level generalization.

In the SV detection task, CMSV remained highly competitive on held-out chr13–22, although the best-performing method varied across platforms and sequencing depths. On PacBio CLR data, CMSV showed stable performance and achieved the highest total F1 at 20× and 10× coverage, suggesting that it can effectively retain and integrate SV evidence in noisy long-read data. On PacBio CCS data, the total F1 of CMSV was close to that of the best-performing method, although Sniffles2, cuteSV2, or SVIM performed slightly better at some coverage levels. On ONT data, CMSV maintained relatively high recall, but cuteSV2 or Sniffles2

achieved higher total F1 in most coverage settings. These results indicate that CMSV has a certain degree of generalization ability in the held-out chromosome detection setting, but its stability still varies across sequencing platforms.

In the genotyping task, CMSV showed a more noticeable performance decrease on held-out chr13–22. In particular, on ONT data and low-coverage PacBio CLR data, cuteSV2 or Sniffles2 usually achieved higher total F1. This may be related to platform-specific sequencing error patterns, local alignment uncertainty, and the balance between variant-supporting reads and reference-supporting reads in genotype inference. Compared with detection, genotyping is more sensitive to read-level support evidence and allele balance. Therefore, its performance is more likely to fluctuate on unseen chromosomes and in low-coverage settings.

Supplementary Note S9. Comparison with CNN-Based Hybrid Deep Learning SV Callers

As shown in Figure S1, existing deep learning-based SV callers usually combine CNNs with different contextual modeling modules. For example, INSnet and MAMnet adopt CNN+BiGRU and CNN+BiLSTM architectures, respectively. SVHunter uses a CNN+Transformer architecture, whereas CMSV uses a CNN-Mamba architecture to aggregate cross-subsegment contextual information within each window. To further illustrate the differences between CMSV and these representative methods in terms of input representation, network architecture, and platform applicability, we summarized the INS/DEL detection results of INSnet, MAMnet, SVHunter, and CMSV on CCS 28×, ONT 48×, and CLR 69× datasets in Table S8.

In terms of input representation, these methods also differ from each other. LSnet mainly uses deletion-related features extracted from long-read/short-read alignments or HiFi reads, and is therefore more focused on DEL detection and genotyping. MAMnet extracts feature matrices from long-read alignments and mainly focuses on DEL and insertion (INS) signals. SVHunter represents each genomic site using multiple alignment-derived features and organizes them into fixed-length feature matrices for CNN–Transformer modeling. In contrast, CMSV constructs a strand-separated, 20-channel, position-level feature matrix from BAM alignments. These channels include CIGAR-derived DEL/INS signals, split-read evidence for multiple SV types, read depth, and clipping signals on both strands.

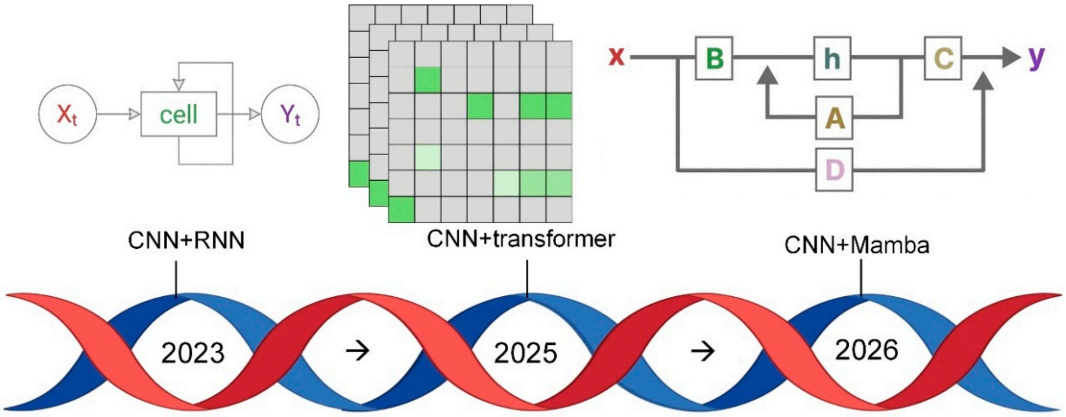

Figure S1. Schematic comparison of CNN-based hybrid deep learning SV calling frameworks.

As shown in Table S8, CMSV showed stable performance in DEL detection and achieved competitive F1 scores across the CCS, ONT, and CLR platforms. This suggests that the CNN–Mamba architecture can effectively integrate deletion-related alignment evidence. For INS detection, CMSV also performed well on CCS and ONT data. In particular, it achieved high recall on ONT data, indicating strong candidate-variant retention and contextual modeling ability in noisy long-read data. Although the best-performing model varied across platforms

and SV types, CMSV maintained stable and competitive detection performance across multiple representative datasets. Overall, this comparison shows that CNN–Mamba can serve as an effective window-level contextual modeling module for integrating multi-channel SV evidence signals and providing reliable candidate-region detection in a hybrid SV calling framework.

Table S8. Comparison of CNN-based hybrid SV callers.

Tool	Network architecture	platform	INS			DEL		
			p	r	F1	p	R	F1
INSnet	CNN + BiGRU	CCS 28×<br>ONT48×<br>CLR 69×	0.9215<br>0.915<br>0.9346	0.922<br>0.8585<br>0.9346	0.9218<br>0.8793<br>0.9247			
MAMnet	CNN + BiLSTM	CCS 28×<br>ONT48×<br>CLR 69×	0.9497<br>0.949<br>0.9697	0.921<br>0.8936<br>0.9062	0.935<br>0.9204<br>0.937	0.966<br>0.947<br>0.9766	0.934<br>0.902<br>0.9463	0.9497<br>0.924<br>0.9614
SVHunter	CNN + Transformer	CCS 28×<br>ONT48×<br>CLR 69×	0.9356<br>0.8850<br>0.9475	0.9340<br>0.8881<br>0.9024	0.9348<br>0.8865<br>0.9244	0.9655<br>0.8992<br>0.9802	0.9405<br>0.9686<br>0.9667	0.9528<br>0.9326<br>0.9735
CMSV	CNN + Mamba	CCS 28×<br>ONT48×<br>CLR 69×	0.9401<br>0.9008<br>0.9428	0.9356<br>0.9595<br>0.8900	0.9378<br>0.9292<br>0.9156	0.9656<br>0.9541<br>0.9811	0.9405<br>0.9793<br>0.9691	0.9529<br>0.9666<br>0.9751

Supplementary Note S10. Sensitivity Analysis of the Heterozygous Balance Factor on HG002 PacBio CCS Genotyping

We performed a sensitivity analysis on the HG002 PacBio CCS 28× dataset aligned to the GRCh38 reference genome. In this experiment, the heterozygous balance factor b was set to 0.5, 0.6, 0.7, and 0.8, respectively, while all other parameters and the upstream SV calling results were kept unchanged.

In the current implementation of CMSV, the default value of b is 0.5. This setting corresponds

to a balanced contribution of variant-supporting reads and reference-supporting reads under a simple diploid heterozygous genotype model. In other words, when adaptive allele-balance modeling related to sequencing platform, SV type, local repeat context, alignment quality, or coverage is not further introduced, $b = 0.5$ is a relatively neutral default setting. Values of $b > 0.5$ indicate a stronger contribution from reference-supporting reads under the heterozygous model, whereas values of $b < 0.5$ would indicate a stronger contribution from variant-supporting reads. Since b is only used for genotype likelihood calculation, this sensitivity analysis mainly focuses on genotype-level metrics.

In genotype-level evaluation, we assessed genotype consistency only on variants that were successfully matched between the callset and the truth set. A variant was considered genotype-correct only when the predicted genotype exactly matched the truth genotype. Ungenotyped calls, such as ./., were not counted as correct genotype matches. Therefore, GT F1 reflects exact genotype consistency among matched variants, rather than only evaluating whether a variant was successfully detected.

Table S9. Sensitivity analysis of the heterozygous balance factor b for CMSV genotyping.

b	GT Concordance	GT Precision	GT Recall	GT F1
0.5	0.9817	0.9215	0.9341	0.9278
0.6	0.9804	0.9203	0.9328	0.9265
0.7	0.9753	0.9156	0.9280	0.9217
0.8	0.9589	0.9002	0.9124	0.9062

As shown in Table S9, $b = 0.5$ achieved the highest GT concordance and GT F1, with values of 0.9817 and 0.9278, respectively. As b increased from 0.5 to 0.8, GT concordance decreased from 0.9817 to 0.9589, and GT F1 decreased from 0.9278 to 0.9062. These results support using $b = 0.5$ as the default setting in the current implementation of CMSV.

Table S10. Per-genotype performance at the default setting *b* = 0.5.

Genotype class	TP	FP	FN	Precision	Recall	F1
0/1	4845	102	62	0.9794	0.9874	0.9834
1/1	3952	52	102	0.9870	0.9748	0.9809

To further examine whether CMSV exhibits systematic heterozygous-to-homozygous or homozygous-to-heterozygous errors, we separately summarized the performance of heterozygous 0/1 and homozygous-alternative 1/1 genotypes under the default setting b = 0.5. The per-genotype F1 values were 0.9834 for 0/1 and 0.9809 for 1/1, indicating that CMSV does not show a strong systematic bias toward either heterozygous or homozygous-alternative genotypes under the default setting.

Table S11. Benchmark Results of detection on HG002 PacBio CCS Dataset（hs37d5）

Dataset	Tool	DEL			INS			Total		
		**Prec**	**Rec**	**F1**	**Prec**	**Rec**	**F1**	**Prec**	**Rec**	**F1**
PacBio CCS 28×	CMSV	0.9656	0.9405	0.9529	0.9401	0.9356	0.9378	0.9467	0.9377	0.9422
	cuteSV2	0.9589	0.9457	0.9523	0.9344	0.9530	0.9436	0.8829	0.9498	0.9151
	Sniffles2	0.9609	0.9362	0.9484	0.9181	0.9430	0.9304	0.9082	0.9400	0.9239
	SVIM	0.9619	0.9390	0.9503	0.9096	0.9230	0.9162	0.8974	0.9300	0.9134
	SVision	0.9442	0.9462	0.9452	0.8559	0.9427	0.8972	0.8842	0.9442	0.9132
PacBio CCS 10×	CMSV	0.9669	0.9077	0.9363	0.9431	0.8762	0.9084	0.9494	0.8900	0.9187
	cuteSV2	0.9624	0.9095	0.9352	0.9388	0.9107	0.9245	0.9007	0.9102	0.9054
	Sniffles2	0.9633	0.8807	0.9201	0.9152	0.8844	0.8995	0.9138	0.8828	0.8980
	SVIM	0.9559	0.9095	0.9321	0.8971	0.8960	0.8966	0.8892	0.9019	0.8955
	SVision	0.9518	0.9080	0.9294	0.8850	0.8982	0.8916	0.9078	0.9025	0.9051
PacBio CCS 5×	CMSV	0.9610	0.8506	0.9024	0.9438	0.7605	0.8423	0.9479	0.7999	0.8677
	cuteSV2	0.9467	0.8883	0.9166	0.8931	0.8886	0.8909	0.7098	0.8885	0.7891
	Sniffles2	0.9655	0.7337	0.8338	0.9261	0.7464	0.8266	0.9254	0.7409	0.8229
	SVIM	0.9640	0.7466	0.8415	0.9216	0.7427	0.8225	0.9136	0.7444	0.8204
	SVision	0.9276	0.8850	0.9058	0.8230	0.8811	0.8511	0.8559	0.8828	0.8691

Table S12. Benchmark Results of detection on HG002 PacBio CLR Dataset (hs37d5)

Dataset	Tool	DEL			INS			Total		
		Prec	Rec	F1	Prec	Rec	F1	Prec	Rec	F1
PacBio CLR 69×	CMSV	0.9811	0.9691	0.9751	0.9428	0.8900	0.9156	0.9052	0.9247	0.9148
	cuteSV2	0.9824	0.9690	0.9757	0.9500	0.9144	0.9318	0.8831	0.9382	0.9098
	Sniffles2	0.9790	0.9557	0.9672	0.6762	0.8807	0.7650	0.7442	0.9134	0.8202
	SVIM	0.9844	0.9612	0.9726	0.9608	0.7692	0.8544	0.8719	0.8528	0.8623
	SVision	0.9732	0.9614	**0.9673**	0.9403	0.8679	0.9026	0.9115	0.9086	0.9100
PacBio CLR 35×	CMSV	0.9834	0.9507	0.9668	0.9518	0.8385	0.8916	0.9179	0.8876	0.9025
	cuteSV2	0.9832	0.9483	0.9655	0.9524	0.8822	0.9160	0.8844	0.9110	0.8975
	Sniffles2	0.9783	0.9436	0.9606	0.6006	0.8614	0.7078	0.6907	0.8972	0.7805
	SVIM	0.9814	0.9669	0.9741	0.9427	0.8104	0.8715	0.8254	0.8785	0.8511
	SVision	0.9665	0.9538	0.9601	0.9220	0.8624	0.8912	0.9115	0.9086	0.9100
PacBio CLR 20×	CMSV	0.9834	0.9077	0.9440	0.9542	0.7489	0.8392	0.9221	0.8185	0.8672
	cuteSV2	0.9865	0.9035	0.9432	0.9530	0.8194	0.8811	0.8890	0.8560	0.8722
	Sniffles2	0.9783	0.9128	0.9444	0.5225	0.8306	0.6415	0.6286	0.8664	0.7286
	SVIM	0.9818	0.9240	0.9520	0.9456	0.7540	0.8390	0.8470	0.8280	0.8374
	SVision	0.9740	0.9026	0.9369	0.9257	0.7962	0.8561	0.9113	0.8425	0.8756
PacBio CLR 10×	CMSV	0.9663	0.8632	0.9118	0.9659	0.5575	0.7069	0.9173	0.6914	0.7885
	cuteSV2	0.9855	0.7599	0.8581	0.9407	0.6505	0.7691	0.8854	0.6982	0.7807
	Sniffles2	0.9720	0.8354	0.8986	0.3400	0.7392	0.4658	0.4602	0.7811	0.5792
	SVIM	0.9828	0.7757	0.8670	0.9502	0.5930	0.7303	0.8594	0.6725	0.7546
	SVision	0.9819	0.7476	0.8488	0.8654	0.6358	0.7331	0.8953	0.6845	0.7758
PacBio CLR 5×	CMSV	0.7722	0.7675	0.7698	0.9024	0.3939	0.5484	0.7708	0.5575	0.6470
	cuteSV2	0.9777	0.6566	0.7856	0.6665	0.5244	0.5870	0.7298	0.5776	0.6449
	Sniffles2	0.9744	0.6175	0.7560	0.3353	0.5149	0.4061	0.4667	0.5596	0.5090
	SVIM	0.9734	0.6542	0.7825	0.9382	0.4912	0.6448	0.8110	0.5622	0.6641
	SVision	0.9639	0.6292	0.7614	0.3373	0.5217	0.4098	0.4830	0.5685	0.5222

Table S13. Benchmark Results of detection on HG002 Oxford Nanopore Dataset (hs37d5)

Dataset	Tool	DEL			INS			Total		
		**Prec**	**Rec**	**F1**	**Prec**	**Rec**	**F1**	**Prec**	**Rec**	**F1**
ONT 48×	CMSV	0.9541	0.9793	0.9666	0.9008	0.9595	0.9292	0.8985	0.9682	0.9320
	cuteSV2	0.9531	0.9771	0.9650	0.9125	0.9125	0.9125	0.9005	0.9407	0.9202
	Sniffles2	0.9162	0.9636	0.9393	0.8821	0.8888	0.8855	0.8737	0.9214	0.8969
	SVIM	0.9112	0.9705	0.9399	0.8675	0.8822	0.8748	0.8670	0.9207	0.8930
	SVision	0.9253	0.9669	0.9456	0.7374	0.8616	0.7947	0.8080	0.9075	0.8549
ONT 20×	CMSV	0.9534	0.9643	0.9588	0.9102	0.9305	0.9202	0.9036	0.9453	0.9240
	cuteSV2	0.9484	0.9450	0.9467	0.9075	0.8920	0.8996	0.8950	0.9151	0.9049
	Sniffles2	0.8942	0.9497	0.9211	0.8220	0.8733	0.8469	0.8335	0.9066	0.8685
	SVIM	0.8749	0.9581	0.9146	0.8174	0.8741	0.8448	0.8221	0.9107	0.8641
	SVision	0.8681	0.9574	0.9105	0.6749	0.8649	0.7582	0.7458	0.9052	0.8178
ONT 10×	CMSV	0.8691	0.9142	0.8911	0.9154	0.8688	0.8915	0.8710	0.8887	0.8797
	cuteSV2	0.9396	0.8564	0.8961	0.9085	0.8082	0.8553	0.8921	0.8292	0.8595
	Sniffles2	0.8758	0.9038	0.8896	0.7953	0.8280	0.8113	0.8148	0.8610	0.8373
	SVIM	0.8679	0.8778	0.8728	0.8184	0.7769	0.7971	0.8262	0.8209	0.8235
	SVision	0.8966	0.8719	0.8841	0.7259	0.7558	0.7405	0.7914	0.8063	0.7988
ONT 5×	CMSV	0.6744	0.7638	0.7163	0.9219	0.7171	0.8067	0.7727	0.7376	0.7547
	cuteSV2	0.9026	0.7392	0.8128	0.8868	0.6911	0.7768	0.8632	0.7121	0.7804
	Sniffles2	0.8802	0.7402	0.8041	0.8064	0.6806	0.7382	0.8272	0.7066	0.7621
	SVIM	0.8186	0.7578	0.7870	0.7833	0.6709	0.7228	0.7837	0.7087	0.7443
	SVision	0.8319	0.7473	0.7874	0.7028	0.6476	0.6741	0.7528	0.6910	0.7206

Table S14. Benchmark Results of Genotyping on HG002 PacBio CCS Dataset（hs37d5）

Dataset	Tool	DEL			INS			Total		
		Prec	Rec	F1	Prec	Rec	F1	Prec	Rec	F1
PacBio CCS 28×	CMSV	0.9484	0.9397	0.9440	0.9239	0.9275	0.9257	0.9294	0.9328	0.9311
	cuteSV2	0.9477	0.9107	0.9288	0.9277	0.9241	0.9259	0.9101	0.9183	0.9142
	Sniffles2	0.9467	0.9224	0.9344	0.8848	0.9089	0.8967	0.8838	0.9147	0.8990
	SVIM	0.9507	0.9281	0.9393	0.8600	0.8727	0.8663	0.8654	0.8968	0.8808
	SVision	0.9206	0.9226	0.9216	0.8141	0.8967	0.8534	0.8503	0.9080	0.8782
PacBio CCS 10×	CMSV	0.9343	0.9048	0.9193	0.9099	0.8682	0.8886	0.9158	0.8842	0.8997
	cuteSV2	0.8876	0.8009	0.8420	0.8845	0.8234	0.8529	0.8631	0.9136	0.8876
	Sniffles2	0.9161	0.8376	0.8751	0.8585	0.8297	0.8438	0.8624	0.8331	0.8475
	SVIM	0.8513	0.8100	0.8301	0.7676	0.7666	0.7671	0.7745	0.7855	0.7800
	SVision	0.8917	0.8507	0.8707	0.7999	0.8118	0.8058	0.8336	0.8288	0.8312
PacBio CCS 5×	CMSV	0.8653	0.8368	0.8508	0.8716	0.7432	0.8023	0.8640	0.7834	0.8217
	cuteSV2	0.8024	0.6566	0.7222	0.8313	0.7207	0.7720	0.7967	0.6928	0.7411
	Sniffles2	0.8709	0.6618	0.7521	0.8354	0.6733	0.7456	0.8347	0.6683	0.7423
	SVIM	0.5929	0.4592	0.5175	0.5561	0.4482	0.4963	0.5559	0.4530	0.4992
	SVision	0.6301	0.6011	0.6152	0.5220	0.5588	0.5398	0.5596	0.5772	0.5683

Table S15. Benchmark Results of Genotyping on HG002 PacBio CLR Dataset (hs37d5)

Dataset	Tool	DEL			INS			Total		
		Prec	Rec	F1	Prec	Rec	F1	Prec	Rec	F1
PacBio CLR 69×	CMSV	0.9358	0.9675	0.9514	0.9176	0.9008	0.9091	0.8710	0.9297	0.8994
	cuteSV2	0.9664	0.9533	0.9598	0.9261	0.8914	0.9084	0.8645	0.9184	0.8906
	Sniffles2	0.9627	0.9397	0.9511	0.6322	0.8234	0.7152	0.7122	0.8741	0.7849
	SVIM	0.9590	0.9364	0.9476	0.7627	0.6106	0.6782	0.7694	0.7525	0.7608
	SVision	0.8946	0.8838	0.8892	0.8166	0.7538	0.7839	0.8129	0.8104	0.8117
PacBio CLR 35×	CMSV	0.9339	0.9477	0.9407	0.9163	0.8516	0.8828	0.8767	0.8934	0.8850
	cuteSV2	0.9600	0.9259	0.9427	0.9161	0.8486	0.8810	0.8565	0.8823	0.8692
	Sniffles2	0.9553	0.9214	0.9381	0.5529	0.7929	0.6515	0.6535	0.8489	0.7385
	SVIM	0.9497	0.9357	0.9427	0.7956	0.6839	0.7356	0.7456	0.7936	0.7688
	SVision	0.8793	0.8678	0.8735	0.7947	0.7433	0.7681	0.7903	0.7975	0.7939
PacBio CLR 20×	CMSV	0.9114	0.9026	0.9070	0.9059	0.7605	0.8269	0.8640	0.8218	0.8424
	cuteSV2	0.9464	0.8669	0.9049	0.8983	0.7723	0.8306	0.8449	0.8135	0.8289
	Sniffles2	0.9416	0.8785	0.9090	0.4721	0.7505	0.5796	0.5849	0.8062	0.6780
	SVIM	0.9426	0.8871	0.9140	0.8034	0.6406	0.7128	0.7651	0.7480	0.7564
	SVision	0.8861	0.8211	0.8524	0.8058	0.6931	0.7452	0.8100	0.7489	0.7782
PacBio CLR 10×	CMSV	0.8339	0.8452	0.8395	0.8614	0.5526	0.6733	0.8016	0.6761	0.7335
	cuteSV2	0.8965	0.6914	0.7807	0.8305	0.5742	0.6790	0.7929	0.6252	0.6992
	Sniffles2	0.8900	0.7649	0.8227	0.2925	0.6360	0.4007	0.4078	0.6921	0.5132
	SVIM	0.8862	0.6995	0.7818	0.7571	0.4724	0.5818	0.7300	0.5713	0.6410
	SVision	0.8461	0.6442	0.7315	0.6936	0.5096	0.5875	0.7432	0.5682	0.6440
PacBio CLR 5×	CMSV	0.5694	0.7099	0.6319	0.7494	0.3679	0.4936	0.5956	0.5036	0.5458
	cuteSV2	0.8109	0.5363	0.6456	0.5465	0.4300	0.4813	0.6018	0.4763	0.5317
	Sniffles2	0.8301	0.5261	0.6440	0.2743	0.4212	0.3322	0.3894	0.4669	0.4246
	SVIM	0.4745	0.3189	0.3814	0.3703	0.1939	0.2545	0.3582	0.2483	0.2933
	SVision	0.5910	0.3858	0.4669	0.1771	0.2738	0.2151	0.2740	0.3226	0.2963

Table S16. Benchmark Results of Genotyping on HG002 Oxford Nanopore Dataset（hs37d5）

Dataset	Tool	DEL			INS			Total		
		Prec	Rec	F1	Prec	Rec	F1	Prec	Rec	F1
ONT 48×	CMSV	0.9219	0.9786	0.9494	0.8740	0.9303	0.9013	0.8707	0.9517	0.9094
	cuteSV2	0.9403	0.9640	0.9520	0.9010	0.9010	0.9010	0.8888	0.9284	0.9082
	Sniffles2	0.9042	0.9509	0.9270	0.8253	0.8315	0.8284	0.8378	0.8835	0.8601
	SVIM	0.8989	0.9574	0.9272	0.7044	0.7163	0.7103	0.7734	0.8213	0.7966
	SVision	0.8870	0.9269	0.9065	0.5863	0.6850	0.6318	0.7037	0.7904	0.7445
ONT 20×	CMSV	0.9171	0.9629	0.9395	0.8749	0.9074	0.8909	0.8670	0.9318	0.8983
	cuteSV2	0.9242	0.9209	0.9226	0.8721	0.8572	0.8646	0.8656	0.8850	0.8752
	Sniffles2	0.8780	0.9326	0.9045	0.7558	0.8030	0.7787	0.7901	0.8595	0.8233
	SVIM	0.8623	0.9443	0.9014	0.6613	0.7073	0.6835	0.7316	0.8105	0.7691
	SVision	0.8281	0.9133	0.8686	0.5303	0.6795	0.5957	0.6438	0.7814	0.7059
ONT 10×	CMSV	0.8049	0.9083	0.8535	0.8423	0.8522	0.8472	0.8023	0.8768	0.8379
	cuteSV2	0.8798	0.8019	0.8390	0.8275	0.7361	0.7792	0.8228	0.7648	0.7927
	Sniffles2	0.8378	0.8645	0.8509	0.6963	0.7249	0.7103	0.7435	0.7857	0.7640
	SVIM	0.8321	0.8416	0.8368	0.6481	0.6152	0.6312	0.7184	0.7138	0.7161
	SVision	0.8366	0.8135	0.8249	0.5649	0.5882	0.5763	0.6736	0.6863	0.6799
ONT 5×	CMSV	0.5989	0.7429	0.6632	0.8113	0.6983	0.7506	0.6825	0.7178	0.6998
	cuteSV2	0.7755	0.6352	0.6984	0.7357	0.5733	0.6444	0.7276	0.6002	0.6578
	Sniffles2	0.7833	0.6587	0.7157	0.6619	0.5586	0.6059	0.7050	0.6022	0.6496
	SVIM	0.4901	0.4537	0.4712	0.3911	0.3350	0.3609	0.4276	0.3867	0.4061
	SVision	0.6047	0.5432	0.5723	0.4364	0.4021	0.4185	0.5050	0.4635	0.4834

Table S17. Benchmark Results of detection on HG002 PacBio CCS Dataset（GRCh38）

Dataset	Tool	DEL			INS			Total		
		Prec	Rec	F1	Prec	Rec	F1	Prec	Rec	F1
PacBio CCS 28×	CMSV	0.9583	0.9606	0.9595	0.9357	0.9456	0.9406	0.9391	0.9522	0.9456
	cuteSV2	0.9677	0.9480	0.9577	0.9322	0.9400	0.9361	0.9448	0.9435	0.9441
	Sniffles2	0.9560	0.9548	0.9554	0.9283	0.9520	0.9400	0.9392	0.9532	0.9462
	SVIM	0.9513	0.9603	0.9557	0.8970	0.9468	0.9212	0.9096	0.9527	0.9307
PacBio CCS 10×	CMSV	0.9585	0.9307	0.9444	0.9364	0.8875	0.9113	0.9399	0.9065	0.9229
	cuteSV2	0.9660	0.8941	0.9287	0.9305	0.8802	0.9046	0.9432	0.8863	0.9138
	Sniffles2	0.9602	0.8962	0.9271	0.9324	0.8789	0.9049	0.9430	0.8865	0.9139
	SVIM	0.9511	0.9279	0.9393	0.8936	0.9106	0.9021	0.9104	0.9182	0.9143
PacBio CCS 5×	CMSV	0.9544	0.8701	0.9103	0.9352	0.7789	0.8499	0.9385	0.8191	0.8747
	cuteSV2	0.9602	0.8154	0.8819	0.9286	0.8167	0.8690	0.9381	0.8161	0.8729
	Sniffles2	0.9604	0.7508	0.8428	0.9324	0.7232	0.8146	0.9431	0.7353	0.8264
	SVIM	0.9359	0.8910	0.9129	0.8697	0.8785	0.8741	0.8861	0.8840	0.8850

Table S18. Benchmark Results of detection on HG002 PacBio CLR Dataset (GRCh38)

Dataset	Tool	DEL			INS			Total		
		Prec	Rec	F1	Prec	Rec	F1	Prec	Rec	F1
PacBio CLR 69×	CMSV	0.9681	0.9389	0.9533	0.9247	0.9126	0.9186	0.9357	0.9242	0.9299
	cuteSV2	0.9733	0.8948	0.9324	0.9096	0.9069	0.9083	0.9345	0.9016	0.9177
	Sniffles2	0.9737	0.9274	0.9500	0.8863	0.9197	0.9027	0.9216	0.9231	0.9224
	SVIM	0.9747	0.9097	0.9411	0.9253	0.8641	0.8936	0.9440	0.8841	0.9131
PacBio CLR 35×	CMSV	0.9690	0.9070	0.9370	0.9312	0.8521	0.8899	0.9419	0.8763	0.9079
	cuteSV2	0.9737	0.8664	0.9169	0.9224	0.8562	0.8881	0.9416	0.8607	0.8993
	Sniffles2	0.9746	0.8981	0.9348	0.8557	0.8770	0.8662	0.9032	0.8863	0.8947
	SVIM	0.9726	0.8801	0.9241	0.9125	0.8316	0.8702	0.9359	0.8529	0.8925
PacBio CLR 20×	CMSV	0.9626	0.8766	0.9176	0.9291	0.7618	0.8372	0.9387	0.8124	0.8710
	cuteSV2	0.9795	0.7671	0.8604	0.9353	0.7205	0.8139	0.9519	0.7409	0.8333
	Sniffles2	0.9762	0.8326	0.8987	0.7297	0.8004	0.7634	0.8216	0.8145	0.8181
	SVIM	0.9753	0.7643	0.8570	0.9313	0.7133	0.8078	0.9489	0.7356	0.8288
PacBio CLR 10×	CMSV	0.9398	0.7542	0.8368	0.9203	0.5313	0.6737	0.9158	0.6294	0.7461
	cuteSV2	0.9817	0.5835	0.7319	0.9194	0.5100	0.6561	0.9437	0.5422	0.6887
	Sniffles2	0.9746	0.6979	0.8133	0.5288	0.6499	0.5831	0.6670	0.6710	0.6690
	SVIM	0.9820	0.5678	0.7196	0.9420	0.5312	0.6793	0.9572	0.5473	0.6964
PacBio CLR 5×	CMSV	0.6107	0.6839	0.6452	0.7232	0.3511	0.4727	0.6303	0.4977	0.5562
	cuteSV2	0.9751	0.4624	0.6273	0.5968	0.3972	0.4769	0.7278	0.4258	0.5372
	Sniffles2	0.9818	0.4199	0.5882	0.5547	0.3785	0.4500	0.6936	0.3966	0.5047
	SVIM	0.9794	0.4496	0.6163	0.9150	0.4256	0.5810	0.9403	0.4361	0.5959

Table S19. Benchmark Results of detection on HG002 ONT Dataset (GRCh38)

Dataset	Tool	DEL			INS			Total		
		Prec	Rec	F1	Prec	Rec	F1	Prec	Rec	F1
ONT 48×	CMSV	0.9499	0.9515	0.9507	0.8980	0.9473	0.9220	0.8984	0.9681	0.9302
	cuteSV2	0.9579	0.9352	0.9464	0.9330	0.9149	0.9238	0.9180	0.9363	0.9238
	Sniffles2	0.9504	0.9376	0.9439	0.9075	0.9134	0.9104	0.9204	0.9240	0.9222
	SVIM	0.9500	0.9435	0.9467	0.8750	0.8914	0.8831	0.8993	0.9143	0.9067
ONT 20×	CMSV	0.9534	0.9640	0.9588	0.9102	0.9305	0.9202	0.9036	0.9453	0.9240
	cuteSV2	0.9542	0.9104	0.9318	0.9304	0.8983	0.9140	0.9335	0.9036	0.9183
	Sniffles2	0.9487	0.9231	0.9358	0.9014	0.8951	0.8983	0.9156	0.9074	0.9115
	SVIM	0.9421	0.9340	0.9380	0.8269	0.8872	0.8560	0.8678	0.9077	0.8873
ONT 10×	CMSV	0.8788	0.9000	0.8893	0.9048	0.8578	0.8807	0.8668	0.8764	0.8716
	cuteSV2	0.9587	0.8350	0.8926	0.9356	0.8205	0.8743	0.9376	0.8269	0.8788
	Sniffles2	0.9522	0.8487	0.8975	0.9084	0.8583	0.8826	0.9221	0.8289	0.8730
	SVIM	0.9465	0.8738	0.9087	0.8332	0.8034	0.8180	0.8753	0.8342	0.8543
ONT 5×	CMSV	0.7388	0.7426	0.7407	0.9131	0.7029	0.7943	0.8037	0.7204	0.7598
	cuteSV2	0.9370	0.7210	0.8150	0.9250	0.7218	0.8108	0.9214	0.7214	0.8092
	Sniffles2	0.9389	0.7307	0.8218	0.8980	0.6988	0.7860	0.9105	0.7128	0.7987
	SVIM	0.8776	0.7541	0.8112	0.8190	0.6977	0.7535	0.8380	0.7225	0.7760

Table S20. Benchmark Results of Genotyping on HG002 PacBio CCS Dataset (GRCh38)

Dataset	Tool	DEL			INS			Total		
		Prec	Rec	F1	Prec	Rec	F1	Prec	Rec	F1
PacBio CCS 28×	CMSV	0.9422	0.9600	0.9510	0.9178	0.9446	0.9310	0.9221	0.9514	0.9365
	cuteSV2	0.9447	0.9468	0.9458	0.9187	0.9392	0.9288	0.9272	0.9425	0.9348
	Sniffles2	0.9422	0.9542	0.9482	0.8201	0.9460	0.8786	0.8719	0.9498	0.9092
	SVIM	0.9375	0.9597	0.9485	0.8459	0.9438	0.8922	0.8749	0.9509	0.9113
PacBio CCS 10×	CMSV	0.9324	0.9289	0.9306	0.9025	0.8837	0.8930	0.9097	0.9037	0.9067
	cuteSV2	0.8830	0.8853	0.8841	0.8718	0.8731	0.8724	0.8741	0.8784	0.8762
	Sniffles2	0.9212	0.8923	0.9065	0.7951	0.8609	0.8267	0.8487	0.8754	0.8619
	SVIM	0.9024	0.9243	0.9132	0.8067	0.9019	0.8516	0.8404	0.9120	0.8747
PacBio CCS 5×	CMSV	0.8652	0.8586	0.8619	0.8521	0.7625	0.8048	0.8530	0.8045	0.8281
	cuteSV2	0.8068	0.7877	0.7971	0.8155	0.7964	0.8058	0.8082	0.7927	0.8004
	Sniffles2	0.8666	0.7311	0.7931	0.7577	0.6798	0.7166	0.8043	0.7032	0.7504
	SVIM	0.6702	0.8541	0.7511	0.5573	0.8225	0.6644	0.5973	0.8370	0.6971

Table S21. Benchmark Results of Genotyping on HG002 PacBio CLR Dataset (GRCh38)

Dataset	Tool	DEL			INS			Total		
		Prec	Rec	F1	Prec	Rec	F1	Prec	Rec	F1
PacBio CLR 69×	CMSV	0.9330	0.9367	0.9349	0.8835	0.9089	0.8960	0.8975	0.9212	0.9092
	cuteSV2	0.9617	0.8937	0.9264	0.8889	0.9050	0.8969	0.9176	0.9000	0.9087
	Sniffles2	0.9516	0.9259	0.9386	0.7016	0.9006	0.7888	0.8050	0.9129	0.8556
	SVIM	0.9357	0.8822	0.9082	0.8108	0.8478	0.8289	0.8629	0.8746	0.8687
PacBio CLR 35×	CMSV	0.9324	0.9037	0.9178	0.8727	0.8437	0.8580	0.8934	0.8705	0.8818
	cuteSV2	0.9503	0.8636	0.9049	0.8826	0.8507	0.8663	0.9089	0.8564	0.8819
	Sniffles2	0.9413	0.8949	0.9175	0.6583	0.8458	0.7404	0.7737	0.8697	0.8189
	SVIM	0.9318	0.8756	0.9028	0.8027	0.8129	0.8077	0.8565	0.8414	0.8489
PacBio CLR 20×	CMSV	0.9138	0.8709	0.8918	0.8340	0.7417	0.7852	0.8657	0.7997	0.8314
	cuteSV2	0.9372	0.7592	0.8388	0.8579	0.7027	0.7726	0.8903	0.7279	0.8009
	Sniffles2	0.9238	0.8248	0.8715	0.5380	0.7473	0.6256	0.6827	0.7849	0.7303
	SVIM	0.9279	0.7552	0.8327	0.8197	0.6865	0.7472	0.8660	0.7175	0.7848
PacBio CLR 10×	CMSV	0.8528	0.7357	0.7900	0.7613	0.4839	0.5917	0.7963	0.5963	0.6820
	cuteSV2	0.8556	0.5497	0.6694	0.7733	0.4667	0.5821	0.8073	0.5033	0.6200
	Sniffles2	0.8696	0.6733	0.7590	0.3658	0.5622	0.4432	0.5224	0.6149	0.5649
	SVIM	0.8307	0.5264	0.6445	0.7155	0.4625	0.5618	0.7646	0.4913	0.5982
PacBio CLR 5×	CMSV	0.4104	0.5925	0.4849	0.5666	0.2977	0.3903	0.4512	0.4150	0.4323
	cuteSV2	0.8036	0.4148	0.5472	0.4681	0.3407	0.3943	0.5846	0.3733	0.4556
	Sniffles2	0.8015	0.3714	0.5076	0.3845	0.2968	0.3350	0.5205	0.3303	0.4042
	SVIM	0.4423	0.2695	0.3350	0.3632	0.2273	0.2796	0.3965	0.2459	0.3036

Table S22. Benchmark Results of Genotyping on HG002 ONT Dataset (GRCh38)

Dataset	Tool	DEL			INS			Total		
		Prec	Rec	F1	Prec	Rec	F1	Prec	Rec	F1
ONT 48×	CMSV	0.9221	0.9786	0.9495	0.8082	0.9550	0.8755	0.8337	0.9658	0.8949
	cuteSV2	0.9431	0.9343	0.9387	0.9220	0.9220	0.9140	0.9300	0.9229	0.9233
	Sniffles2	0.9444	0.9419	0.9432	0.8516	0.9354	0.8916	0.8886	0.9384	0.9128
	SVIM	0.9212	0.9418	0.9314	0.7235	0.8716	0.7907	0.8017	0.9048	0.8501
ONT 20×	CMSV	0.9171	0.9629	0.9395	0.8168	0.9232	0.8667	0.8369	0.9412	0.8860
	cuteSV2	0.9314	0.9084	0.9197	0.8983	0.8950	0.8966	0.9056	0.9009	0.9032
	Sniffles2	0.9383	0.9257	0.9320	0.8173	0.9006	0.8569	0.8668	0.9123	0.8890
	SVIM	0.9094	0.9318	0.9205	0.6840	0.8668	0.7646	0.7720	0.8975	0.8300
ONT 10×	CMSV	0.8163	0.8932	0.8530	0.7654	0.8362	0.7992	0.7658	0.8624	0.8112
	cuteSV2	0.9001	0.8261	0.8615	0.8619	0.8081	0.8341	0.8710	0.8161	0.8427
	Sniffles2	0.9128	0.8613	0.8863	0.7564	0.8142	0.7842	0.8212	0.8363	0.8287
	SVIM	0.8919	0.8671	0.8793	0.6789	0.7690	0.7211	0.7645	0.8147	0.7888
ONT 5×	CMSV	0.6617	0.7209	0.6901	0.7442	0.6585	0.6987	0.6845	0.6869	0.6857
	cuteSV2	0.8233	0.6943	0.7533	0.7875	0.6883	0.7346	0.7954	0.6910	0.7395
	Sniffles2	0.8233	0.6943	0.7533	0.7875	0.6883	0.7346	0.7494	0.6458	0.6937
	SVIM	0.6781	0.7033	0.6905	0.5488	0.6073	0.5766	0.6009	0.6512	0.6250

Table S23. Benchmark Results of detection on HG002 PacBio CCS Dataset (GRCh38 chr13-chr22)

Dataset	Tool	DEL			INS			Total		
		**Prec**	**Rec**	**F1**	**Prec**	**Rec**	**F1**	**Prec**	**Rec**	**F1**
PacBio CCS 28×	CMSV	0.9569	0.9460	0.9514	0.9207	0.9401	0.9303	0.9357	0.9426	0.9391
	cuteSV2	0.9690	0.9523	0.9606	0.9368	0.9395	0.9382	0.9483	0.9452	0.9468
	Sniffles2	0.9580	0.9611	0.9595	0.9320	0.9504	0.9411	0.9421	0.9551	0.9486
	SVIM	0.9538	0.9650	0.9594	0.9071	0.9486	0.9274	0.9172	0.9559	0.9361
PacBio CCS 10×	CMSV	0.9573	0.9164	0.9364	0.9194	0.8871	0.9030	0.9353	0.8995	0.9171
	cuteSV2	0.9687	0.9027	0.9345	0.9358	0.8785	0.9062	0.9476	0.8892	0.9175
	Sniffles2	0.9634	0.9046	0.9331	0.9359	0.8761	0.9050	0.9461	0.8888	0.9166
	SVIM	0.9552	0.9335	0.9442	0.9031	0.9102	0.9066	0.9181	0.9205	0.9193
PacBio CCS 5×	CMSV	0.9545	0.8573	0.9033	0.9187	0.7781	0.8425	0.9343	0.8116	0.8686
	cuteSV2	0.9613	0.8215	0.8859	0.9345	0.8117	0.8688	0.9424	0.8161	0.8747
	Sniffles2	0.9631	0.7544	0.8461	0.9374	0.7234	0.8166	0.9468	0.7372	0.8290
	SVIM	0.9379	0.8975	0.9173	0.8799	0.8772	0.8785	0.8932	0.8862	0.8897

Table S24. Benchmark Results of detection on HG002 PacBio CLR Dataset (GRCh38 chr13-chr22)

Dataset	Tool	DEL			INS			Total		
		Prec	Rec	F1	Prec	Rec	F1	Prec	Rec	F1
PacBio CLR 69×	CMSV	0.9611	0.9252	0.9428	0.9213	0.8960	0.9085	0.9380	0.9084	0.9230
	cuteSV2	0.9755	0.9030	0.9378	0.9109	0.9104	0.9106	0.9366	0.9071	0.9216
	Sniffles2	0.9752	0.9319	0.9530	0.8928	0.9192	0.9058	0.9265	0.9249	0.9257
	SVIM	0.9775	0.9163	0.9459	0.9328	0.8689	0.8997	0.9500	0.8899	0.9190
PacBio CLR 35×	CMSV	0.9623	0.8886	0.9240	0.9290	0.8348	0.8794	0.9433	0.8576	0.8984
	cuteSV2	0.9758	0.8774	0.9240	0.9233	0.8598	0.8904	0.9432	0.8676	0.9038
	Sniffles2	0.9770	0.9082	0.9413	0.8554	0.8769	0.8660	0.9046	0.8908	0.8977
	SVIM	0.9761	0.8881	0.9300	0.9211	0.8341	0.8754	0.9429	0.8581	0.8985
PacBio CLR 20×	CMSV	0.9600	0.8564	0.9052	0.9229	0.7404	0.8217	0.9396	0.7895	0.8580
	cuteSV2	0.9808	0.7794	0.8686	0.9354	0.7253	0.8170	0.9532	0.7493	0.8391
	Sniffles2	0.9786	0.8465	0.9078	0.7295	0.8024	0.7642	0.8246	0.8220	0.8233
	SVIM	0.9780	0.7777	0.8664	0.9403	0.7198	0.8154	0.9553	0.7456	0.8375
PacBio CLR 10×	CMSV	0.9355	0.7067	0.8052	0.9138	0.5070	0.6522	0.9246	0.5915	0.7214
	cuteSV2	0.9828	0.5931	0.7398	0.9210	0.5175	0.6627	0.9463	0.5511	0.6966
	Sniffles2	0.9795	0.7148	0.8265	0.5229	0.6580	0.5827	0.6664	0.6833	0.6747
	SVIM	0.9857	0.5805	0.7307	0.9451	0.5409	0.6880	0.9610	0.5585	0.7064
PacBio CLR 5×	CMSV	0.6161	0.6536	0.6343	0.7244	0.3202	0.4441	0.6553	0.4612	0.5414
	cuteSV2	0.9760	0.4750	0.6390	0.5909	0.4077	0.4825	0.7257	0.4376	0.5460
	Sniffles2	0.9839	0.4364	0.6046	0.5495	0.3921	0.4576	0.6925	0.4118	0.5165
	SVIM	0.9827	0.4620	0.6286	0.9215	0.4391	0.5948	0.9465	0.4493	0.6094

Table S25. Benchmark Results of detection on HG002 ONT Dataset (GRCh38 chr13-chr22)

Dataset	Tool	DEL			INS			Total		
		**Prec**	**Rec**	**F1**	**Prec**	**Rec**	**F1**	**Prec**	**Rec**	**F1**
ONT 48×	CMSV	0.9422	0.9365	0.9393	0.8608	0.9464	0.9016	0.8932	0.9422	0.9171
	cuteSV2	0.9596	0.9413	0.9504	0.9399	0.9133	0.9264	**0.9351**	**0.9196**	**0.9273**
	Sniffles2	**0.9496**	**0.9432**	**0.9464**	**0.9142**	**0.9128**	**0.9135**	**0.9252**	**0.9250**	**0.9251**
	SVIM	0.9520	0.9465	0.9492	0.8890	0.8922	0.8906	0.9093	0.9163	0.9128
ONT 20×	CMSV	0.9407	0.9243	0.9324	0.8768	0.9126	0.8944	0.9029	0.9176	0.9102
	cuteSV2	0.9554	0.9169	0.9358	0.9371	0.8974	0.9168	0.9380	0.9061	0.9218
	Sniffles2	**0.9539**	**0.9285**	**0.9410**	**0.9142**	**0.8867**	**0.9002**	**0.9267**	**0.9038**	**0.9151**
	SVIM	0.9454	0.9374	0.9413	0.8419	0.8891	0.8649	0.8796	0.9106	0.8948
ONT 10×	CMSV	0.8854	0.8808	0.8831	0.8833	0.8450	0.8638	0.8842	0.8601	0.8720
	cuteSV2	0.9599	0.8391	0.8954	0.9414	0.8172	0.8749	0.9417	0.8269	0.8806
	Sniffles2	**0.9465**	**0.8596**	**0.9010**	**0.9313**	**0.8427**	**0.8848**	**0.9193**	**0.8366**	**0.8760**
	SVIM	0.9507	0.8764	0.9120	0.8465	0.8078	0.8267	0.8854	0.8383	0.8612
ONT 5×	CMSV	0.7186	0.7224	0.7205	0.8909	0.6926	0.7793	0.8071	0.7052	0.7527
	cuteSV2	0.9387	0.7258	0.8187	0.9326	0.7185	0.8117	0.9263	0.7218	0.8114
	Sniffles2	**0.9646**	**0.7261**	**0.8285**	**0.9295**	**0.6840**	**0.7881**	**0.9392**	**0.7013**	**0.8030**
	SVIM	0.8789	0.7609	0.8157	0.8372	0.7011	0.7631	0.8496	0.7277	0.7839

Table S26. Benchmark Results of Genotyping on HG002 PacBio CCS Dataset (GRCh38 chr13-chr22)

Dataset	Tool	DEL			INS			Total		
		Prec	Rec	F1	Prec	Rec	F1	Prec	Rec	F1
PacBio CCS 28×	CMSV	0.9322	0.9217	0.9269	0.9038	0.9228	0.9132	0.9156	0.9223	0.9190
	cuteSV2	0.9485	0.9513	0.9499	0.9231	0.9387	0.9308	0.9317	0.9443	0.9379
	Sniffles2	0.9466	0.9606	0.9536	0.8324	0.9448	0.8851	0.8815	0.9522	0.9155
	SVIM	0.9429	0.9646	0.9536	0.8607	0.9460	0.9013	0.8866	0.9544	0.9193
PacBio CCS 10×	CMSV	0.9127	0.8738	0.8928	0.8870	0.8559	0.8711	0.8978	0.8635	0.8803
	cuteSV2	0.8907	0.8950	0.8928	0.8780	0.8715	0.8747	0.8810	0.8818	0.8814
	Sniffles2	0.9312	0.9016	0.9162	0.8100	0.8596	0.8340	0.8621	0.8793	0.8706
	SVIM	0.9097	0.9304	0.9199	0.8204	0.9020	0.8593	0.8523	0.9149	0.8825
PacBio CCS 5×	CMSV	0.8440	0.7581	0.7987	0.8524	0.7219	0.7818	0.8487	0.7372	0.7890
	cuteSV2	0.8166	0.7964	0.8064	0.8200	0.7909	0.8052	0.8151	0.7933	0.8041
	Sniffles2	0.8778	0.7368	0.8011	0.7692	0.6822	0.7231	0.8160	0.7074	0.7578
	SVIM	0.6755	0.8631	0.7578	0.5606	0.8198	0.6658	0.6024	0.8401	0.7017

Table S27. Benchmark Results of Genotyping on HG002 PacBio CLR Dataset (GRCh38 chr13-chr22)

Dataset	Tool	DEL			INS			Total		
		Prec	Rec	F1	Prec	Rec	F1	Prec	Rec	F1
PacBio CLR 69×	CMSV	0.9123	0.8782	0.8949	0.8879	0.8635	0.8755	0.8981	0.8697	0.8837
	cuteSV2	0.9653	0.9021	0.9326	0.8909	0.9086	0.8996	0.9208	0.9057	0.9132
	Sniffles2	0.9586	0.9308	0.9445	0.7241	0.9023	0.8034	0.8227	0.9162	0.8669
	SVIM	0.9312	0.9013	0.9160	0.8238	0.8540	0.8387	0.8748	0.8816	0.8782
PacBio CLR 35×	CMSV	0.9105	0.8407	0.8742	0.8737	0.7851	0.8270	0.8895	0.8086	0.8471
	cuteSV2	0.9542	0.8749	0.9128	0.8859	0.8547	0.8701	0.9128	0.8638	0.8876
	Sniffles2	0.9462	0.9055	0.9254	0.6753	0.8490	0.7523	0.7876	0.8766	0.8297
	SVIM	0.9390	0.8841	0.9108	0.8165	0.8167	0.8166	0.8686	0.8478	0.8581
PacBio CLR 20×	CMSV	0.9024	0.8050	0.8510	0.8307	0.6665	0.7396	0.8629	0.7251	0.7880
	cuteSV2	0.9408	0.7721	0.8481	0.8600	0.7082	0.7768	0.8939	0.7371	0.8080
	Sniffles2	0.9317	0.8400	0.8835	0.5538	0.7551	0.6390	0.6989	0.7965	0.7445
	SVIM	0.9347	0.7698	0.8443	0.8368	0.6957	0.7598	0.8793	0.7295	0.7975
PacBio CLR 10×	CMSV	0.8491	0.6414	0.7308	0.7552	0.4190	0.5390	0.8021	0.5131	0.6258
	cuteSV2	0.8575	0.5598	0.6774	0.7731	0.4738	0.5875	0.8093	0.5122	0.6274
	Sniffles2	0.8791	0.6922	0.7745	0.3737	0.5790	0.4542	0.5329	0.6330	0.5787
	SVIM	0.8364	0.5400	0.6563	0.7305	0.4766	0.5769	0.7764	0.5054	0.6123
PacBio CLR 5×	CMSV	0.4053	0.4299	0.4172	0.5758	0.2545	0.3529	0.4671	0.3287	0.3858
	cuteSV2	0.8053	0.4275	0.5585	0.4641	0.3509	0.3996	0.5838	0.3850	0.4640
	Sniffles2	0.7966	0.3854	0.5194	0.3872	0.3125	0.3458	0.5222	0.3455	0.4159
	SVIM	0.4438	0.2794	0.3429	0.3777	0.2429	0.2956	0.4060	0.2592	0.3164

Table S28. Benchmark Results of Genotyping on HG002 ONT Dataset (GRCh38 chr13-chr22)

Dataset	Tool	DEL			INS			Total		
		Prec	Rec	F1	Prec	Rec	F1	Prec	Rec	F1
ONT 48×	CMSV	0.9332	0.9774	0.9548	0.8179	0.9509	0.8794	0.8479	0.9627	0.9017
	cuteSV2	0.9471	0.9405	0.9438	0.9262	0.9085	0.9173	0.9305	0.9249	0.9277
	Sniffles2	0.9477	0.9477	0.9477	0.8580	0.9372	0.8959	0.8944	0.9421	0.9176
	SVIM	0.9279	0.9451	0.9364	0.7433	0.8738	0.8033	0.8181	0.9079	0.8606
ONT 20×	CMSV	0.9279	0.9614	0.9444	0.8280	0.9196	0.8714	0.8521	0.9383	0.8931
	cuteSV2	0.9354	0.9153	0.9253	0.9073	0.8944	0.9008	0.9128	0.9038	0.9083
	Sniffles2	0.9447	0.9329	0.9387	0.8246	0.9008	0.8611	0.8747	0.9159	0.8948
	SVIM	0.9175	0.9356	0.9265	0.7042	0.8703	0.7785	0.7897	0.9014	0.8419
ONT 10×	CMSV	0.8308	0.8914	0.8600	0.7759	0.8261	0.8002	0.7823	0.8554	0.8172
	cuteSV2	0.9046	0.8309	0.8662	0.8684	0.8048	0.8354	0.8771	0.8165	0.8457
	Sniffles2	0.9164	0.8663	0.8906	0.7678	0.8146	0.7905	0.8304	0.8391	0.8347
	SVIM	0.8993	0.8702	0.8845	0.6950	0.7753	0.7330	0.7783	0.8201	0.7987
ONT 5×	CMSV	0.6693	0.7173	0.6925	0.7564	0.6553	0.7022	0.6984	0.6832	0.6907
	cuteSV2	0.8275	0.7000	0.7585	0.7937	0.6848	0.7352	0.8010	0.6917	0.7423
	Sniffles2	0.8509	0.6953	0.7653	0.6990	0.6158	0.6548	0.7640	0.6531	0.7042
	SVIM	0.6855	0.7128	0.6989	0.5647	0.6127	0.5877	0.6147	0.6591	0.6361

Table S29. Benchmark results on SURVIVOR simulated CLR detection

Tool	SV Type	TP-call	TP-base	Total prediction	Precision	Recall	F1
	DEL	964	964	1046	0.9216	0.9797	0.9498
	INS	841	837	1220	0.6893	0.8238	0.7506
CMSV	DUP	971	970	973	0.9979	0.9700	0.9838
	INV	979	979	981	0.9980	0.9790	0.9884
	TRA	3497	1690	3568	0.9801	0.8450	0.9075
	DEL	905	905	938	0.9648	0.9197	0.9417
	INS	684	684	721	0.9487	0.6732	0.7876
SVIM	DUP	968	968	968	1.0000	0.9680	0.9837
	INV	810	810	810	1.0000	0.8100	0.8950
	TRA	4124	1309	4124	1.0000	0.6545	0.7912
	DEL	968	968	992	0.9758	0.9837	0.9798
	INS	965	896	1306	0.7389	0.8819	0.8041
SVision	DUP	700	700	700	1.0000	0.7000	0.8235
	INV	199	199	2737	0.0727	0.1990	0.1065
	TRA	0	0	0	0.0000	0.0000	0.0000
	DEL	969	968	978	0.9908	0.9837	0.9873
	INS	1027	985	2446	0.4199	0.9695	0.5860
Sniffles2	DUP	950	949	951	0.9989	0.9490	0.9733
	INV	990	988	1127	0.8784	0.9880	0.9300
	TRA	4086	1618	4090	0.9990	0.8090	0.8940
	DEL	950	950	985	0.9645	0.9654	0.9650
	INS	588	588	614	0.9577	0.5787	0.7215
cuteSV2	DUP	952	952	953	0.9990	0.9520	0.9749
	INV	1885	982	1947	0.9682	0.9820	0.9750
	TRA	1849	1156	1851	0.9989	0.5780	0.7323

Table S30. Benchmark results on SURVIVOR simulated ONT detection

Tool	SV Type	TP-call	TP-base	Total prediction	Precision	Recall	F1
CMSV	DEL	972	972	996	0.9759	0.9878	0.9818
	INS	1003	983	1638	0.6123	0.9675	0.7500
	DUP	966	966	971	0.9949	0.9660	0.9802
	INV	985	984	989	0.9960	0.9840	0.9899
	TRA	3657	1766	3724	0.9820	0.8830	0.9299
SVIM	DEL	964	964	985	0.9787	0.9797	0.9792
	INS	942	942	984	0.9573	0.9272	0.9420
	DUP	958	958	958	1.0000	0.9580	0.9785
	INV	895	895	895	1.0000	0.8950	0.9446
	TRA	5322	1555	5326	0.9992	0.7775	0.8745
SVision	DEL	976	975	990	0.9859	0.9909	0.9883
	INS	1163	1003	1591	0.7310	0.9872	0.8400
	DUP	913	911	913	1.0000	0.9110	0.9534
	INV	252	251	2873	0.0877	0.2510	0.1300
	TRA	0	0	0	0.0000	0.0000	0.0000
Sniffles2	DEL	975	974	993	0.9819	0.9898	0.9858
	INS	1008	1006	2776	0.3631	0.9902	0.5314
	DUP	955	954	956	0.9990	0.9540	0.9760
	INV	993	988	1309	0.7586	0.9880	0.8582
	TRA	3770	1644	3774	0.9989	0.8220	0.9019
cuteSV2	DEL	973	972	998	0.9749	0.9878	0.9813
	INS	884	884	1005	0.8796	0.8701	0.8748
	DUP	952	952	953	0.9990	0.9520	0.9749
	INV	1963	989	2175	0.9025	0.9890	0.9438
	TRA	2244	1292	2246	0.9991	0.6460	0.7847

Table S31. Benchmark results on VISOR simulated CLR detection

SV Type	Tool	TP-call	TP-base	Total prediction	Precision	Recall	F1
Deletion	CMSV	5907	6280	6002	0.9842	0.9002	0.9403
	cuteSV2	5761	5885	5952	0.9679	0.8432	0.9013
	Sniffles2	6082	6191	6308	0.9642	0.8871	0.9240
	SVIM	6323	6461	6606	0.9572	0.9258	0.9412
	SVision	6403	6433	6658	0.9617	0.9218	0.9413
Insertion	CMSV	7999	7947	8170	0.9791	0.8220	0.8937
	cuteSV2	7454	7605	8067	0.9240	0.7866	0.8498
	Sniffles2	7576	7734	8176	0.9266	0.8000	0.8587
	SVIM	9083	8893	10921	0.8317	0.9198	0.8735
	SVision	9090	8985	11347	0.8011	0.9294	0.8605

Table S32. Benchmark results on VISOR simulated ONT detection

SV Type	Tool	TP-call	TP-base	Total prediction	Precision	Recall	F1
Deletion	CMSV	5885	6247	5981	0.9839	0.8955	0.9376
	cuteSV2	5775	5916	5999	0.9627	0.8477	0.9015
	Sniffles2	6115	6206	6345	0.9638	0.8892	0.9250
	SVIM	6314	6448	6648	0.9498	0.9239	0.9367
	SVision	6423	6442	6707	0.9576	0.9231	0.9400
Insertion	CMSV	7963	7899	8137	0.9786	0.8170	0.8905
	cuteSV2	7359	7521	8103	0.9082	0.7779	0.8380
	Sniffles2	7434	7587	8045	0.9241	0.7848	0.8488
	SVIM	9111	8879	11317	0.8051	0.9184	0.8580
	SVision	9086	8895	11705	0.7762	0.9200	0.8420

Table S33. Benchmark results on VISOR simulated CLR genotyping

SV Type	Tool	TP-call	TP-base	Total prediction	Precision	Recall	F1
Deletion	CMSV	5728	6088	6002	0.9543	0.8727	0.9117
	cuteSV2	5699	5816	5952	0.9575	0.8334	0.8912
	Sniffles2	5985	6093	6308	0.9488	0.8730	0.9093
	SVIM	5983	6085	6606	0.9057	0.8680	0.8864
	SVision	5856	5911	6658	0.8795	0.8470	0.8629
Insertion	CMSV	7206	7318	8170	0.8820	0.7569	0.8147
	cuteSV2	7243	7378	8067	0.8979	0.7631	0.8250
	Sniffles2	7165	7302	8176	0.8763	0.7553	0.8113
	SVIM	7053	7068	10921	0.6458	0.7311	0.6858
	SVision	6854	6913	11347	0.6040	0.7150	0.6548

Table S34. Benchmark results on VISOR simulated ONT genotyping

SV Type	Tool	TP-call	TP-base	Total prediction	Precision	Recall	F1
Deletion	CMSV	5759	6098	5981	0.9629	0.8741	0.9164
	cuteSV2	5689	5824	5999	0.9483	0.8345	0.8878
	Sniffles2	5981	6076	6345	0.9426	0.8706	0.9052
	SVIM	5955	6033	6648	0.8533	0.8645	0.8589
	SVision	5884	5927	6707	0.8773	0.8493	0.8631
Insertion	CMSV	6995	7126	8137	0.8597	0.7371	0.7937
	cuteSV2	7123	7253	8103	0.8791	0.7502	0.8096
	Sniffles2	6935	7075	9668	0.7173	0.7318	0.7245
	SVIM	6813	6805	11317	0.6020	0.7039	0.6490
	SVision	6593	6637	11705	0.5633	0.6865	0.6188

Table S35. Detection performance on HG00514 and NA19240 datasets at 10× coverage

	**DEL/INS**	**CMSV**	**SVIM**	**cuteSV2**	**Sniffles2**
**HG00514**	Precision	0.7389/0.7950	0.3904/0.6272	0.6733/0.7334	0.6738/0.5117
	Recall	0.3064/0.1750	0.0710/0.0658	0.2817/0.2021	0.2219/0.1574
	F1	0.4331/0.2869	0.1201/0.1192	**0.3972/0.3169**	0.3339/0.2408
**NA19240**	Precision	0.8034/0.7789	0.5372/0.6653	0.7508/0.7232	0.7213/0.5475
	Recall	0.3038/0.1570	0.0735/0.0604	0.2641/0.1791	0.2248/0.1485
	F1	0.4409/0.2614	0.1293/0.1108	**0.3908/0.2871**	0.3427/0.2336

Table S36. Detection performance on HG00514 and NA19240 datasets at 5× coverage

	**DEL/INS**	**CMSV**	**SVIM**	**cuteSV2**	**Sniffles2**
**HG00514**	Precision	0.5350/0.7205	0.3541/0.5444	0.6655/0.6186	0.6757/0.5024
	Recall	0.2452/0.1370	0.0739/0.0689	0.2315/0.1615	0.1713/0.1240
	F1	0.3362/0.2302	0.1222/0.1224	**0.3435/0.2561**	0.2733/0.1989
**NA19240**	Precision	0.5684/0.6664	0.4957/0.5972	0.7566/0.6153	0.7399/0.5475
	Recall	0.2380/0.1092	0.0620/0.0508	0.1892/0.1221	0.1442/0.0982
	F1	0.3355/0.1876	0.1103/0.0936	**0.3027/0.2038**	0.2414/0.1665

Table S37. Mendelian discordance rate (MDR) results stratified by SV type in the Ashkenazi trio

SV type	CMSV（CLR）			cuteSV2（CLR）			Sniffles2（CLR）			SVIM（CLR）		
	Total calls	Not in parents	MDR (%)	Total calls	Not in parents	MDR (%)	Total calls	Not in parents	MDR (%)	Total calls	Not in parents	MDR (%)
DEL	7681	487	6.34	11848	1029	8.69	8007	522	6.52	13064	1273	9.74
INS	9687	1402	14.47	18263	4303	23.56	14364	4052	28.21	16583	2178	13.13
DUP	1535	1211	78.89	1463	1341	91.66	399	390	97.74	1694	1554	91.74
INV	32	15	46.88	312	196	62.82	160	90	56.25	50	40	80.00
BND	454	410	90.31	1566	597	38.12	1016	595	58.56	2980	2044	68.59
Total	19389	3525	18.18	33452	7466	22.32	23946	5649	23.59	34371	7089	20.62
SV type	CMSV（CCS）			cuteSV2（CCS）			Sniffles2（CCS）			SVIM（CCS）		
	Total calls	Not in parents	MDR (%)	Total calls	Not in parents	MDR (%)	Total calls	Not in parents	MDR (%)	Total calls	Not in parents	MDR (%)
DEL	8708	361	4.15	15877	1312	8.26	10350	659	6.37	15347	491	3.20
INS	12895	481	3.73	19454	2031	10.44	15679	1050	6.70	21291	1315	6.18
DUP	133	58	43.61	422	199	47.16	7	4	57.14	184	66	35.87
INV	25	1	4.00	166	40	24.10	53	13	24.53	22	0	0.00
BND	546	366	67.03	1171	335	28.61	1207	790	65.45	1104	144	13.04
Total	22307	1267	5.68	37090	3917	10.56	27296	2516	9.22	37948	2016	5.31
SV type	CMSV（ONT）			cuteSV2（ONT）			Sniffles2（ONT）			SVIM（ONT）		
	Total calls	Not in parents	MDR (%)	Total calls	Not in parents	MDR (%)	Total calls	Not in parents	MDR (%)	Total calls	Not in parents	MDR (%)
DEL	9128	525	5.75	29859	5941	19.9	30056	5313	17.68	41278	7856	19.03
INS	12964	800	6.17	20424	2071	10.14	18210	2329	12.79	24293	2072	8.53
DUP	1180	536	45.42	726	157	21.63	172	30	17.44	430	97	22.56
INV	43	7	16.28	154	12	7.79	106	12	11.32	26	1	3.85
BND	3040	2074	68.22	1467	208	14.18	777	347	44.66	1296	162	12.50
Total	26355	3942	14.96	52630	8389	15.94	49321	8031	16.28	67323	10188	15.13

Table S38. Mendelian discordance rate (MDR) results stratified by SV type in the Chinese trio

SV type	CMSV（CLR）			cuteSV2（CLR）			Sniffles2（CLR）			SVIM（CLR）		
	Total calls	Not in parents	MDR (%)	Total calls	Not in parents	MDR (%)	Total calls	Not in parents	MDR (%)	Total calls	Not in parents	MDR (%)
DEL	9425	1187	12.59	15699	2060	13.12	15252	1545	10.13	16949	1842	10.87
INS	14098	4824	34.22	54702	33559	61.35	37260	17190	46.14	20341	3652	17.95
DUP	403	116	28.78	475	104	21.89	87	20	22.99	265	47	17.74
INV	41	5	12.20	227	24	10.57	97	19	19.59	25	3	12.00
BND	934	624	66.81	1595	357	22.38	1091	574	52.61	372	83	22.31
Total	**24901**	**6756**	**27.13**	72698	36104	49.66	53787	19348	35.97	37952	5627	14.83
SV type	CMSV（CCS）			cuteSV2（CCS）			Sniffles2（CCS）			SVIM（CCS）		
	Total calls	Not in parents	MDR (%)	Total calls	Not in parents	MDR (%)	Total calls	Not in parents	MDR (%)	Total calls	Not in parents	MDR (%)
DEL	9544	356	3.73	14327	633	4.42	16137	583	3.61	17339	567	3.27
INS	14134	521	3.69	17977	1324	7.36	21431	1117	5.21	23894	1425	5.96
DUP	444	70	15.77	434	69	15.90	66	14	21.21	573	92	16.06
INV	80	6	7.50	236	10	4.24	109	19	17.43	37	1	2.70
BND	1902	424	22.29	1072	226	21.08	381	212	55.64	1416	317	22.39
Total	**26104**	**1377**	**5.28%**	34046	2262	6.64	38124	1945	5.10	43259	2402	5.55
SV type	CMSV（ONT）			cuteSV2（ONT）			Sniffles2（ONT）			SVIM（ONT）		
	Total calls	Not in parents	MDR (%)	Total calls	Not in parents	MDR (%)	Total calls	Not in parents	MDR (%)	Total calls	Not in parents	MDR (%)
DEL	9582	686	7.16	18191	1947	10.70	17909	2092	11.68	21656	2264	10.45
INS	13677	727	5.32	19575	4596	8.15	19028	1822	9.58	25256	1852	7.33
DUP	1059	250	23.61	587	107	18.23	152	27	17.76	371	67	18.06
INV	74	8	10.81	168	18	10.71	98	10	10.20	32	1	3.12
BND	4384	891	20.32	755	147	19.47	168	72	42.86	134	48	35.82
Total	**28776**	**2562**	**8.90**	39276	3815	9.71	37355	4023	10.77	47449	4232	8.92

Table S39. Mendelian-inheritance error rate comparison on the GIAB Ashkenazi Jewish trio

SV type	CMSV（CLR）			cuteSV2（CLR）			Sniffles2（CLR）			SVIM（CLR）		
	Total calls	Mendelian err	MIER (%)	Total calls	Mendelian err	MIER (%)	Total calls	Mendelian err	MIER (%)	Total calls	Mendelian err	MIER (%)
DEL	3478	44	1.27	5450	169	3.10	3808	100	2.63	5898	184	3.12
INS	2819	89	3.16	6517	232	3.56	5504	395	7018	7107	433	6.09
DUP	30	0	0.00	20	3	15.00	3	0	0.00	16	2	12.50
INV	16	1	6.25	35	1	2.86	23	0	0.00	5	0	0.00
BND	14	0	0.00	543	29	5.34	261	11	4.21	——	——	——
Total	6357	134	2.11	12565	434	3.45	9592	506	5.28	13026	619	4.75

SV type	CMSV（CCS）			cuteSV2（CCS）			Sniffles2（CCS）			SVIM（CCS）		
	Total calls	Mendelian err	MIER (%)	Total calls	Mendelian err	MIER (%)	Total calls	Mendelian err	MIER (%)	Total calls	Mendelian err	MIER (%)
DEL	4625	54	1.17	7419	172	2.32	5319	87	1.64	8041	72	0.90
INS	7353	121	1.65	9099	159	1.75	8176	194	2.37	10446	142	1.36
DUP	40	8	20.00	66	9	13.64	0	0	0.00	43	0	0.00
INV	20	1	5.00	54	4	7.41	17	0	0.00	18	0	0.00
BND	60	0	0.00	565	40	7.08	346	29	8.38	——	——	——
Total	12098	184	1.52	17203	384	2.23	13858	310	2.24	18548	214	1.15

SV type	CMSV（ONT）			cuteSV2（ONT）			Sniffles2（ONT）			SVIM（ONT）		
	Total calls	Mendelian err	MIER (%)	Total calls	Mendelian err	MIER (%)	Total calls	Mendelian err	MIER (%)	Total calls	Mendelian err	MIER (%)
DEL	4859	87	1.79	14087	333	2.36	15154	165	1.09	20299	561	2.76
INS	7378	307	4.16	7952	260	3.27	8694	222	2.55	10828	459	4.24
DUP	325	28	8.62	250	13	5.20	65	0	0.00	37	3	8.11
INV	27	0	0.00	56	0	0.00	58	0	0.00	17	0	0.00
BND	382	10	2.62	852	50	5.87	304	13	4.28	——	——	——
Total	12971	432	3.33	23197	656	2.83	24275	400	1.65	31181	1023	3.28

Table S40. Mendelian-inheritance error rate comparison on the GIAB Chinese human trio

SV type	CMSV（CLR）			cuteSV2（CLR）			Sniffles2（CLR）			SVIM（CLR）		
	Total calls	Mendelian err	MIER (%)	Total calls	Mendelian err	MIER (%)	Total calls	Mendelian err	MIER (%)	Total calls	Mendelian err	MIER (%)
DEL	4520	49	1.08	7415	154	2.08	7610	88	1.16	8319	143	1.72
INS	4087	95	2.32	8355	220	2.63	9720	152	1.56	8381	314	3.75
DUP	156	7	4.49	85	4	4.71	29	0	0.00	6	0	0.00
INV	23	3	13.04	87	4	4.60	37	0	0.00	17	0	0.00
BND	112	0	0.00	602	21	3.49	328	17	5.18	——	——	——
Total	8898	154	1.73	16544	403	2.44	17724	257	1.45	16723	457	2.73

SV type	CMSV（CCS）			cuteSV2（CCS）			Sniffles2（CCS）			SVIM（CCS）		
	Total calls	Mendelian err	MIER (%)	Total calls	Mendelian err	MIER (%)	Total calls	Mendelian err	MIER (%)	Total calls	Mendelian err	MIER (%)
DEL	5396	60	1.11	7713	101	1.31	8882	54	0.61	9666	104	1.08
INS	8278	111	1.34	8949	102	1.14	11223	126	1.12	12236	198	1.62
DUP	219	18	8.22	100	3	3.00	12	0	0.00	4	0	0.00
INV	53	5	9.43	89	2	2.25	44	0	0.00	19	0	0.00
BND	452	12	2.65	401	19	4.74	94	3	3.19	——	——	——
Total	14398	206	1.43	17252	227	1.32	20255	183	0.90	21925	302	1.38

SV type	CMSV（ONT）			cuteSV2（ONT）			Sniffles2（ONT）			SVIM（ONT）		
	Total calls	Mendelian err	MIER (%)	Total calls	Mendelian err	MIER (%)	Total calls	Mendelian err	MIER (%)	Total calls	Mendelian err	MIER (%)
DEL	5217	50	0.96	8721	241	2.76	8890	69	0.78	10801	217	2.01
INS	8017	254	3.17	8731	220	2.52	9741	189	1.94	11943	491	4.11
DUP	402	24	5.97	234	12	5.13	60	1	1.67	6	0	0.00
INV	54	1	1.85	69	3	4.35	50	0	0.00	21	0	0.00
BND	614	20	3.26	332	19	5.72	51	2	3.92	——	——	——
Total	14304	349	2.44	18087	495	2.74	18792	261	1.39	22771	708	3.11

Table S41. Resource consumption of different SV callers.

Platform	Coverage	Tool	Threads	Elapsed runtime (min)	Max memory (GB)
CCS	5×	SVIM	1	8.14	0.26
		Sniffles2	16	0.52	0.53
		cuteSV2	16	2.41	0.84
		SVision	16	211.00	3.71
		CMSV	16	61.45	79.43
	10×	SVIM	1	10.65	0.35
		Sniffles2	16	0.89	0.55
		cuteSV2	16	3.33	0.85
		SVision	16	576.48	12.87
		CMSV	16	62.40	116.00
ONT	5×	SVIM	1	11.04	0.62
		Sniffles2	16	2.04	0.58
		cuteSV2	16	2.46	0.85
		SVision	16	507.15	3.52
		CMSV	16	56.98	80.44
	10×	SVIM	1	23.06	0.79
		Sniffles2	16	2.88	0.65
		cuteSV2	16	4.61	0.89
		SVision	16	1183.34	5.56
		CMSV	16	78.72	127.95
CLR	5×	SVIM	1	23.70	0.78
		Sniffles2	16	1.53	0.63
		cuteSV2	16	2.04	0.79
		SVision	16	353.43	3.49
		CMSV	16	43.42	86.39
	10×	SVIM	1	41.93	1.45
		Sniffles2	16	3.18	0.78
		cuteSV2	16	4.07	1.24
		SVision	16	1739.08	3.33
		CMSV	16	55.57	123.79

Runtime and maximum memory consumption were measured using GNU /usr/bin/time -v. Runtime was taken from “Elapsed (wall clock) time” and converted into minutes. Maximum memory consumption was taken from “Maximum resident set size” and converted to GB using kB / 1024 / 1024, representing the peak host RAM usage.
